# Cuticular hydrocarbons as potential mediators of cryptic species divergence in a mutualistic ant association

**DOI:** 10.1002/ece3.5464

**Published:** 2019-07-21

**Authors:** Juliane Hartke, Philipp P. Sprenger, Jacqueline Sahm, Helena Winterberg, Jérôme Orivel, Hannes Baur, Till Beuerle, Thomas Schmitt, Barbara Feldmeyer, Florian Menzel

**Affiliations:** ^1^ Senckenberg Biodiversity and Climate Research Centre Frankfurt am Main Germany; ^2^ Institute of Organismic and Molecular Evolution Johannes‐Gutenberg‐University Mainz Mainz Germany; ^3^ Department of Animal Ecology and Tropical Biology University of Würzburg Würzburg Germany; ^4^ CNRS, UMR EcoFoG (AgroParisTech, CIRAD, INRA, Université des Antilles, Université de Guyane) Kourou Cedex France; ^5^ Department of Invertebrates Natural History Museum Bern Bern Switzerland; ^6^ Institute of Ecology and Evolution University of Bern Bern Switzerland; ^7^ Institute of Pharmaceutical Biology University of Technology Braunschweig Braunschweig Germany; ^8^ Present address: Department of Evolutionary Animal Ecology University of Bayreuth Bayreuth Germany

**Keywords:** environmental association, integrative taxonomy, niche differentiation, population structure, sexual selection, speciation

## Abstract

Upon advances in sequencing techniques, more and more morphologically identical organisms are identified as cryptic species. Often, mutualistic interactions are proposed as drivers of diversification. Species of the neotropical parabiotic ant association between *Crematogaster levior* and *Camponotus femoratus* are known for highly diverse cuticular hydrocarbon (CHC) profiles, which in insects serve as desiccation barrier but also as communication cues. In the present study, we investigated the association of the ants’ CHC profiles with genotypes and morphological traits, and discovered cryptic species pairs in both genera. To assess putative niche differentiation between the cryptic species, we conducted an environmental association study that included various climate variables, canopy cover, and mutualistic plant species. Although mostly sympatric, the two *Camponotus* species seem to prefer different climate niches. However in the two *Crematogaster* species, we could not detect any differences in niche preference. The strong differentiation in the CHC profiles may thus suggest a possible role during speciation itself either by inducing assortative mating or by reinforcing sexual selection after the speciation event. We did not detect any further niche differences in the environmental parameters tested. Thus, it remains open how the cryptic species avoid competitive exclusion, with scope for further investigations.

## 1 INTRODUCTION

Diversity on earth is reflected in the ongoing discovery of a large number of species every year. Among animals, insects are especially species‐rich and, out of an estimated 5 million species, only about 1 million have been described (Stork, 2018). Finding new species can be challenging due to remote and undiscovered habitats or a high morphological similarity to closely related species. The latter, so‐called cryptic species, are defined as distinct, but morphologically similar species (Bickford et al., 2007). They are often identified coincidentally based on genetic data, chemical profiles, or behavior. The lack of morphological differentiation between cryptic species can be due to recent divergence and too little time for distinct morphological features to evolve (Grundt, Kjølner, Borgen, Rieseberg, & Brochmann, 2006; Gustafson, Kensinger, Bolek, & Luttbeg, 2014), or by selection on morphological stasis (Bickford et al., 2007; Struck et al., 2018). It has also been postulated that taxa, which communicate mating signals via nonvisual cues (e.g., chemicals, vibrations, sounds), are more likely to harbor cryptic species, as morphological differentiation in these taxa is less important than, for example, in some birds, which use visual signals as mating displays (Andersson, 1982; Hudson & Price, 2014).

Given that cryptic species are morphologically alike and often closely related, one would expect them to be ecologically very similar and to exhibit only slight niche differentiation (Violle, Nemergut, Pu, & Jiang, 2011). Already, very subtle ecological divergence in traits like thermal niche or food preferences, as well as spatio‐temporal heterogeneity (e.g., different availability of resources), could allow such species to share the same habitat and avoid competitive exclusion (Gause, 1932; Hardin, 1960; Scriven, Whitehorn, Goulson, & Tinsley, 2016). In ants for example, cryptic species can occur sympatrically, if they inhabit distinct niches, for example, by specializing on different symbiotic fungi (Schultz et al., 2002). Next to the question how cryptic species coexist, it is also often unclear how species boundaries can be maintained between closely related species sharing the same habitat. One proposed mechanism is the expression of phenotypic traits that lead to assortative mating and thus reduce gene flow (Dieckmann & Doebeli, 1999). In this context, phenotypic traits might favor speciation even in sympatry if they are shaped by ecological selection pressures and at the same time induce assortative mating (so‐called “magic traits”), such as color patterns or smell (Nosil, 2012; Servedio, Doorn, Kopp, Frame, & Nosil, 2011; Thibert‐Plante & Gavrilets, 2013).

Species interactions can promote and speed up the emergence of novel phenotypic traits and lead to coevolution and diversification (Guimarães, Jordano, & Thompson, 2011; Hoeksema & Bruna, 2000; Thompson, Schwind, Guimarães, & Friberg, 2013). For mutualisms, adaptive dynamics models predict that if in a population of a mutualistic species certain groups of one species become more attractive and are thus chosen as partners more often, evolutionary branching should occur (i.e., the split into two distinct phenotypic clusters; Doebeli & Dieckmann, 2000). This dimorphism in one mutualistic partner can lead to disruptive selection in the other partner and therefore to a cospeciation event (Doebeli & Dieckmann, 2000). Although strict cospeciation seems rather rare (de Vienne et al., 2013), in mutualisms it was described repeatedly, for example, between arthropods and their endosymbionts (Bolaños et al., 2019; Degnan, Lazarus, Brock, & Wernegreen, 2004; Hosokawa, Kikuchi, Nikoh, Shimada, & Fukatsu, 2006), in specialized ant–plant mutualisms (Chomicki, Ward, & Renner, 2015), and fig‐pollinating wasps and figs (Cruaud et al., 2012; Jousselin et al., 2008). Alternatively, species diversification in mutualisms can also be facilitated by partner switches like in pollination mutualisms (Janz, Nyblom, & Nylin, 2001; Kawakita, Takimura, Terachi, Sota, & Kato, 2004) or ant–plant associations (Quek, Davies, Itino, & Pierce, 2004).

A remarkable example of mutualism is parabioses, which are defined as interactions between two different ant species sharing a nest with separate brood chambers (Menzel, Linsenmair, & Blüthgen, 2008; Orivel, Errard, & Dejean, 1997). Here, we investigate the neotropical ant species *Crematogaster levior* and *Camponotus femoratus* that live parabiotically in so‐called ant gardens and both profit from abilities of their partners (Davidson, 1988; Vantaux, Dejean, Dor, & Orivel, 2007). Although the two species share a common nest and show interspecific tolerance, they keep their own species‐specific cuticular hydrocarbon (CHC) profiles (Emery & Tsutsui, 2013). Previous studies revealed two substantially different chemical phenotypes (or chemotypes) in both *Cr. levior* and *Ca. femoratus*, that otherwise were morphologically and ecologically indistinguishable (Emery & Tsutsui, 2013; Menzel, Orivel, Kaltenpoth, & Schmitt, 2014). CHCs cover the cuticle of basically all terrestrial arthropods. They are the main component of the waxy epicuticular layer, whose primary role is to prevent desiccation (Blomquist & Bagnères, 2010). However, CHCs secondarily evolved several important roles in chemical communication like mediating recognition of mating partners (Thomas & Simmons, 2008), and (in social insects) of nestmates and castes (van Zweden & d'Ettorre, 2010). A CHC profile usually consists of structurally different groups of hydrocarbons, namely straight‐chained *n*‐alkanes, mono‐ or polymethyl‐branched alkanes, and mono‐ or poly‐unsaturated alkenes, in different combinations (Blomquist, 2010). As CHC profiles are usually species‐specific, but similar even between distant populations (Martin, Helanterä, & Drijfhout, 2008), high diversity is unusual within a single species.

In this study, we elucidate the species status of the different chemotypes of both, *Cr. levior* and *Ca. femoratus*, by multiple lines of evidence within the framework of integrative taxonomy (Heethoff, Laumann, Weigmann, & Raspotnig, 2011; Steiner et al., 2018). We compared cuticular hydrocarbons, secondary metabolites, morphological traits and genotypes between different colonies, and find clear evidence for two cryptic species in each of the two genera. Next, we asked whether these cryptic species differ ecologically and conducted an environmental association study including local climate, mutualistic partners, ant garden plants, and canopy cover. Finally, we tested for partner preferences among the mutualistic species.

## 2 MATERIALS AND METHODS

### 2.1 Sampling

We collected parabiotic ants of the species *Crematogaster levior* and *Camponotus femoratus* along an east–west gradient in French Guiana from August to October 2016. The east–west transect in French Guiana coincides with a climatic gradient (i.e., higher precipitation and lower temperatures in the east of the country and vice versa). We only collected ants foraging outside the nests, thereby leaving the colonies intact. To make sure, we sampled different colonies of these polydomous species, and we only collected ants from ant gardens which were at least 20 m apart from each other. In total, we collected 333 colonies from 13 different locations (Table 1). If we could not reach the garden itself, we looked for shared trails or extrafloral nectaries attended by both species. In some of these cases (*n* = 20), we were not able to obtain individuals of *Ca. femoratus*. For each colony collected, we took a GPS point using a Garmin eTrex H personal navigator (Garmin Europe Ltd.), noted plant genera present on the ant gardens (*Philodendron*,* Aechmea*,* Codonanthe*,* Peperomia*, and *Anthurium*), and took a vertical photo of the canopy with a Nikon Coolpix W100 (Nikon GmbH). Samples for genetic and morphological analyses were stored in 99% ethanol.

**Table 1:** Sampling sites with details on sampled and analyzed colonies

Site	Code	#	Latitude	Longitude	Elevation (m)	Number of colonies	Genetically analyzed samples (Cr|Ca)	Chemically analyzed samples (Cr|Ca)
Apatou	AP	1	5.200783	−54.312017	28	16	16|16	16|16
Saint‐Laurent	SL	2	5.463902	−53.997322	63	36	33|29	36|32
Angoulême	AN	3	5.409200	−53.650933	64	1	01|01	01|01
Sinnamary	SI	4	5.352035	−53.077604	45	20	20|20	19|20
Petit Saut	PS	5	5.061213	−52.988772	93	21	19|17	18|18
Paracou	PAR	6	5.265905	−52.933605	41	53	50|47	50|49
Les Nouragues	LN	7	4.039650	−52.673933	63	74	72|60	72|61
Kourou	KO	8	5.083106	−52.643022	23	12	12|10	11|11
Montsinéry	MT	9	4.866000	−52.538483	26	4	04|04	04|04
Cacao	CA	10	4.557416	−52.463067	71	22	21|19	21|20
Cayenne	CAY	11	4.793831	−52.317594	20	6	06|05	06|05
Régina	RE	12	4.181286	−52.131963	82	16	16|13	16|14
Patawa	PAT	13	4.546067	−52.130483	282	52	52|48	52|51

Note

**Figure 1:**
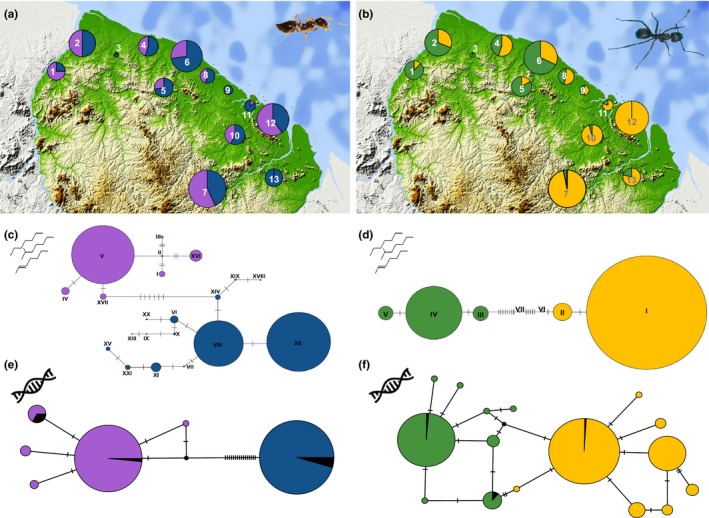
Chemotype and haplotype distribution across French Guiana and their differentiation. (a) Distribution of the cryptic *Cr. levior* species (*Cr. levior* A: blue; *Cr. levior* B: purple). The size of the circles reflects the number of sampled colonies. (b) Distribution of cryptic *Ca. femoratus* species (*Ca. femoratus* PAT: yellow; *Ca. femoratus* PS: green). Numbers in (a) and (b) refer to sampling locations in Table 1. (c) and (d) chemical networks of *Cr. levior* and *Ca. femoratus*, using the same color code. (e) and (f) Haplotype networks (based on COI) using the same color code. Black coloration represents colonies without CHC information. Circles represent chemical types or haplotypes, respectively, and hatch marks indicate the number of character changes between them. Circle sizes reflect the number of colonies per chemical type or haplotype with singletons depicted slightly larger than according to their proportion. Pictures of *Cr. levior* (a) and *Ca. femoratus* (b) (© B. Feldmeyer)

Numbers (#) of sampling sites refer to numbers on the map in Figure 1.

### 2.2 Chemical analyses

To analyze the CHC profiles, we immersed 10 freeze‐killed *Cr. levior* or 5 *Ca. femoratus* workers per colony for 10 min in hexane. In *Cr. levior*, the cuticle contained polar secondary metabolites next to CHCs. These two substance groups were separated by fractionation using SiOH columns (Chromabond, 1 ml/100 mg, Macherey‐Nagel). CHC fractions were eluted with hexane; the polar compounds were eluted with dichloromethane. The samples of polar compounds were dried under a gentle nitrogen stream and redissolved in approximately 50 µl hexane for analysis.

Cuticular hydrocarbons were analyzed using gas chromatography–mass spectrometry (GC‐MS). The gas chromatograph (7890A, Agilent Technologies) was equipped with a Zebron Inferno ZB5‐MS capillary column (length 30 m, Ø 0.25 mm, 0.25 µm coating, Phenomenex), and helium was used as carrier gas with a flow rate of 1.2 ml per minute. The mass spectrometer (5975C, Agilent Technologies) was used with electron ionization (EI) at 70 eV.

For the *Cr. levior* CHC extracts, 4 µl were injected into the GC at 40°C using a PTV (programmed temperature vaporizing) method and this temperature was held constant for 2 min. Thereafter, the oven heated up with 60°C per minute to 200°C and above this temperature with 4°C per minute to 320°C which were kept for 10 min. The PTV method allows a higher injection volume, which was needed because of the presumably lower quantity of the much smaller *Crematogaster* ants. In *Ca. femoratus*, 2 µl of extract was injected at 60°C using the splitless method. The oven heated up with 60°C per minute to 200°C and then with 4°C per minute to 320°C which again were kept constant for 10 min. The same temperature program as for *Camponotus* CHCs was used to analyze the polar compounds of *Cr. levior*. The resulting chromatograms were integrated manually using *MSD ChemStation* (E.02.02.1431, Agilent Technologies).

CHCs were identified using Kovats indices and diagnostic ions (Carlson, Bernier, & Sutton, 1998). We excluded all substances which were not hydrocarbons as well as substances which had proportions less than 0.1% on average or were present in less than 20% of the samples (of the respective chemotype). Because the number of double bonds sometimes differed between colonies, we still included substances with multiple double bonds even if they occurred in less than 20% of the samples if other alkenes of the same chain length were present in other samples.

The polar substances produced by *Cr. levior* were likewise analyzed via GC‐MS as described above. They were aligned based on their mass spectra using a custom database. To investigate the molecular formula of the polar substances, highly concentrated samples of the *Cr. levior* A and B (100 individuals per sample) were analyzed using GC‐EI‐HRMS (gas chromatography coupled with high‐resolution mass spectrometry). The setup we used allows the generation of accurate masses to establish molecular formulae of molecular and fragment ions at ∆m < 3.0 mmu. For GC‐EI‐HRMS, we used an Agilent 6890 gas chromatograph equipped with an analytical column (30 m × 0.25 mm i.d., film thickness 0.25 µm; ZB‐1MS, Phenomenex), helium as carrier gas (1.0 ml/min; constant flow mode), and a temperature program of 100°C (3 min)–10°C/min–320°C (10 min). Injection volume was 1 µl in splitless mode. The gas chromatograph (GC) was coupled directly to a JMS‐T100GC time‐of‐flight (TOF) mass spectrometer (GCAccuTOF, JEOL) in electron ionization (EI) mode at 70 eV. The source and transfer line temperatures were set at 200 and 310°C, respectively. The detector voltage was set at 2050 V. The acquisition mass range was set from *m*/*z* 41 to *m*/*z* 650 with a spectrum recording interval of 0.4 s. The system was tuned with perfluorokerosene to achieve a resolution of 6,000 (full width at half maximum) at *m*/*z* 292.9824. JEOL MassCenterTM workstation software was used for data acquisition and data evaluation.

### 2.3 Statistical analyses—chemical data

In total, we analyzed 322 different *Cr. levior* and 302 *Ca. femoratus* colonies. The colonies were assigned to the CHC chemotypes described previously (Menzel et al., 2014) based on NMDS ordinations (Figure S1).

To check for major differences in the CHC composition, we pooled substances according to their substance class (*n*‐alkanes, mono‐, di‐ and trimethyl alkanes, mono‐unsaturated alkenes, alkadienes, alkatrienes, and methyl‐branched alkenes). We tested whether their abundances (dependent variables) differed between the two chemotypes of either genus (fixed factor) using PERMANOVAs (command *adonis*, R‐package *vegan* [Oksanen et al., 2016]). If a certain substance class was absent from several samples, we added minute normally distributed random numbers (mean: 10^–8^ ± 10^–8^) to the respective class for all samples, as PERMANOVA cannot manage samples with zero distance. This was only the case for alkadienes and methyl‐branched alkenes in *Crematogaster*.

To quantitate the separation of the chemotypes, we adapted the concept of haplotype networks to CHC profiles. As compositional data are continuous, we categorized the profiles based on a principal component analysis (PCA). This method has the advantage that one can quantitate the separation between CHC profiles and display information of multiple PC axes (i.e., more than two dimensions) at the same time and provide a clear visualization of the degree of variation between and within groups. To this end, we firstly performed a PCA based on our CHC data after centered log‐ratio (clr) transformation (Aitchison, 1982; Brückner & Heethoff, 2017). Subsequently, we assigned a number of possible categories to each PC axis based on their eigenvalues (i.e., the number of categories per PC axis equaled its eigenvalue divided by 5 to obtain a “handable” number of axes and distances between samples) and was rounded to two if the eigenvalue was between 10 and 5. PC axes with eigenvalues <5 were not considered. In our case, most of the CHC variation was explained by the first PCs, which is why we only used the first three PCs for the network of *Crematogaster* (explained variance: 58.75%) and the first two PCs for the network of *Camponotus* (explained variance: 73.75%; all other PCs having eigenvalues <5).

Then, the PC loadings for each sample were transformed into distinct categories by dividing the distance of a certain PC loading to the minimum by the whole range of the PC loadings and rounding this value to integer numbers. As a result, we obtained a sequence of categories for each sample, with the length of the character sequence being the number of PC axes used. We used the R‐package *pegas* (Paradis, 2010) with the *haplotype* command to calculate different clusters (chemical types) based on the character sequences. Subsequently, we calculated the (integer) Euclidean distances between samples for each PC axis and summed them up. Networks were then constructed using *haploNet* (package *pegas*).

To find out whether *Cr. levior* populations can be differentiated by their polar metabolites, we visualized ordinations based on Bray–Curtis distance matrices. Additionally, we performed random forest analyses using the *randomForest* package (Liaw & Wiener, 2002) to check whether we could assign the samples to the CHC chemotype based on their polar substances. All statistics were conducted using R version 3.5.0 (R Core Team, 2018).

### 2.4 Morphological measurements

After classification based on the CHC profiles, we measured 30–40 individuals per cryptic species of both genera from independent colonies that were randomly distributed over the different sampling locations (total *N* = 160). As *Ca. femoratus* workers are dimorphic, we took only minors (the smaller caste) for our analyses. All measurements were taken blindly in a random order (per genus) using a Keyence VHX‐2000 digital microscope (Keyence International (Belgium) NV/SA, Urdorf, Switzerland). Thirty specimens of *Cr*. and *Ca*. were photographed and measured twice to assess reliability (= 1—measurement error, see Bartlett & Frost, 2008). In the further analysis, we took the mean of both measurements for those specimens. Variables with reliability <85% were omitted from the analyses (Table S1; Figure S2). For calculating reliability, we used the intraclass correlation coefficient with the function *ICCest* as provided by the R‐package *ICC* (see also Wolak, Fairbairn, & Paulsen, 2012).

We measured 23 characters for *Crematogaster* and 20 characters for *Camponotus* (based on Seifert, 2008; Csösz et al., 2014; and additional criteria). For *Crematogaster*, all measurements were taken under 200‐fold magnification, while for *Camponotus* three different magnifications were used due to their larger body size. We used 100‐fold to measure the mesosoma, 150‐fold for head, legs, and antennae, and 200‐fold magnification for all other characters of *Camponotus*. Measurements were taken using ImageJ (version 1.50e, National Institutes of Health) and the *straight* measure tool. We used an in‐house ImageJ script to convert pixels into µm for each measurement.

We used multivariate ratio analysis (MRA) to analyze our body measurements. MRA comprises a set of tools for analyzing size and shape separately in a multivariate framework (see e.g., Baur & Leuenberger, 2011; Baur et al., 2014; Gebiola et al., 2017 for a detailed description of the application). One of these tools is the shape PCA, which in contrast to a conventional PCA, allows to compare body shape irrespective of *isometric* body size. The effect of allometric variation (e.g., allometric scaling, see Baur & Leuenberger, 2011; Klingenberg, 2016) may then be explored by plotting the first two shape PCs against isometric size. First, we ran a shape PCA for each genus separately. Next, the PCA ratio spectrum, another method of the MRA toolkit, allowed the interpretation of individual shape PCs in terms of ratios. Finally, isometric size was calculated as the geometric mean of all measurements per individual. For calculating the shape PCA, isometric size, and the PCA ratio spectra, we used a slightly modified version of the R script published by Baur et al. (2014). Plots were generated using *ggplot2* (Wickham, 2016).

To statistically test for morphological separation of the cryptic species, we calculated MANOVAs with the first two shape PCs as dependent variables and the species identity as well as sampling location as fixed factors. We used the first two PC axes since they explained 48% and 56.6% of the variance in *Crematogaster* and *Camponotus*, respectively (the cryptic species did not differ in PC3). To compare the isometric size between each species within a genus, we calculated Welch two‐sample *t* tests. Calculation of these statistics was done with the basic functions *MANOVA* and *t.test* provided by R.

### 2.5 COI barcoding

To test for genetic separation, one individual of *Cr. levior* and *Ca. femoratus* of every sampled colony was barcoded at the mitochondrial COI locus. DNA was extracted following the HotSHOT protocol (see Montero‐Pau, Gomez, & Muñoz, 2008). For DNA extraction, two legs of each individual of *Cr. levior* and one leg for *Ca. femoratus*, respectively, were used and DNA fragments of the COI locus (primers: LCO1490, HCO2198) were amplified using the following PCR cycling protocol: 5 min of denaturation at 95°C, followed by 35 cycles of 30 s of denaturation at 95°C, 60 s annealing at 48°C, and 90 s extension at 72°C. This was followed by a final extension step at 72°C for 10 min. For detailed PCR and sequencing reaction mix, see Table S2. Thermocycler conditions for the sequencing reaction were as follows: 1 min of denaturation at 95°C, followed by 30 cycles of 10 s denaturation at 96°C, 10 s of annealing at 50°C, and 2 min extension at 60°C. This was followed by 10 min of final extension at 72°C. Resulting DNA fragments were sequenced on an ABI PRISM 3700 (Thermo Fisher Scientific). Sequences were trimmed and aligned in GENEIOUS v. 10.1.3 using the ClustalW (Thompson, Higgins, & Gibson, 1994) plugin. All sequences were manually checked and curated if necessary. The final alignment had a length of 449 bases.

### 2.6 COI—parsimony networks, phylogeny, and population genetic parameters

Haplotype networks were created for *Cr. levior* and *Ca. femoratus* using the TCS algorithm in PopART v. 1.7 (Leigh & Bryant, 2015). In addition, Bayesian phylogenies were created using MrBayes v. 3.2 (Ronquist et al., 2012) upon identification of the best substitution model (HKY + G for *Crematogaster* and *Camponotus*) with MEGA7 (Kumar, Stecher, Li, Knyaz, & Tamura, 2018). Phylogenetic analyses for both species ran for 13,500,000 generations for *Cr. levior* and 9,020,500 for *Ca. femoratus*, respectively, with a burn‐in of 25%; trees were sampled every 500 generations. Resulting trees were visualized in Archaeopteryx v. 0.992 beta (Han & Zmasek, 2009). Based on networks and phylogenies, *Cr. levior* and *Ca. femoratus* were both separated into two distinct clusters each corresponding to the previously identified chemotypes. Thus, for the following analyses, we treated them as four separate cryptic species and call them *Cr. levior* A and B, as well as *Ca. femoratus* PAT and PS.

To investigate allele frequency differences between the different sampling sites, pairwise *F*_ST_ values were calculated between all population pairs separately for each of the two cryptic species pairs of *Cr. levior* and *Ca. femoratus*, using Arlequin v. 3.5 (Excoffier & Lischer, 2010). In addition, Tajima's D (Tajima, 1989) was calculated as a measure for potential selection.

### 2.7 Nuclear markers for *Camponotus*

Based on the small number of SNPs that separate the two cryptic species of *Ca. femoratus* at the COI locus, we sequenced four additional nuclear loci to obtain more details on the genetic population structure. For *Cr. levior*, we plan to use a PoolSeq approach in a future study to obtain this information on a genome wide basis. In the following, we sequenced one individual per colony from locations with at least three PAT and three PS colonies (max. 12 colonies). In total, 14 unannotated Exon‐primed intron‐crossing (EPIC) primers (Table S3; Ströher, Li, & Pie, 2013) were tested. Four primer pairs (ant.1FR, ant.389FR, ant.1087FR, and ant.1401FR) that amplified and showed variability were sequenced and further analyzed. The PCR master mix was the same as for COI barcoding, except for 0.1 µl of each primer instead of 0.2 µl. Thermocycler conditions were as follows: 5 min of denaturation at 95°C followed by 35 cycles of 1 min of denaturation at 92°C for primer pair 1,087 and 40 cycles for the remaining primer pairs, respectively, 1 min of annealing at 59°C and 2 min extension at 70°C. This was followed by 6 min of final extension at 72°C. For details on the sequencing reaction, see above in the COI section. Forward and reverse sequences were assembled and manually curated. Alignment lengths differed between all loci (ant.1:137 bp, ant.389:239 bp, ant.1087:379 bp, and ant.1401 399 bp = 1,154 bp in total), and so did the number of sequence polymorphisms (ant.1:4 SNPs, ant.389:4 SNPs, ant.1087:5 SNPs, ant.1401:5 SNPs = 18 SNPs in total).

### 2.8 *Camponotus* nuclear markers—parsimony networks and phylogeny

As for COI, we calculated the TCS networks with PopART (Leigh & Bryant, 2015). We furthermore used BEAST v. 2.5 (Bouckaert et al., 2014) to calculate a phylogeny based on all four nuclear markers and the previously obtained COI sequences, comprising all individuals for which each locus was successfully sequenced (*n* = 93). Each locus was tested for the best substitution model in MEGA7 (Kumar et al., 2018). BEAUTi, implemented within the BEAST package, was used to set up specifications for BEAST using StarBEAST2. Based on Akaike's information criterion (AIC), we chose JC69 as best substitution model for nuclear marker ant.1FR and HKY for all others. For all markers, a relaxed log normal clock model was used. Remaining parameters were set to default. BEAST was started with a chain length of 100,000,000, sampling trees every 1,000 generations. The resulting trees were summarized in TreeAnnotator (included in BEAST) with a burn‐in of 20% that was previously established in TRACER v. 1.6 (Rambaut, Drummond, Xie, Baele, & Suchard, 2018). The resulting tree was visualized in Archeaopteryx v. 0.992beta (Han & Zmasek, 2009). In addition, we used STRUCTURE 2.3.4 on the same dataset. The admixture model was used for calculations with a burn‐in period of 10,000 and a number of MCMC repetitions of 1,000,000 for a set number of two populations (*k* = 2).

### 2.9 Ecological and environmental association

Based on chemical and genetic information, we could unambiguously assign each colony to *Cr*. *levior* A or B, or *Ca. femoratus* PAT or PS. First, we tested for nonrandom associations between the two cryptic *Crematogaster* and *Camponotus* species using a chi‐squared test.

Second, we obtained climate data from CHELSA Bioclim variables (Karger et al., 2017), consisting of composed climate data for the years 1979–2013 for the GPS location of every sampled colony. We performed a PCA with all 19 climate variables to reduce the number of variables. Most variance was explained by the first PC axis (76.47%) and was characterized by an inverse relationship of precipitation and temperature variables (i.e., higher precipitation correlates with colder temperatures). A high factor loading coincided with high annual precipitation (mean: 3,137.08 mm; minimum: 1979 mm; and maximum: 4,873 mm) and a low annual mean temperature (mean: 25.6°C; minimum: 24.4°C; and maximum: 26.3°C).

Third, the presence/absence of plant genera on the ant nest was coded as a binomial variable (1 = present and 0 = absent). Canopy cover was estimated in ImageJ: All pictures taken from the canopy above each ant nest were converted to black and white using the *Make binary* command; covered areas were measured using the *Histogram* function. The obtained data were transformed to relative proportions.

For each colony, we created binomial variables of the species for *Crematogaster* (A vs. B) and *Camponotus* (PAT vs. PS). These were used as dependent variables in two binomial generalized linear mixed models with logit link function. As explanatory variables, we used the loading of PC1 from the climate PCA described above, the percentage of canopy covered, the identity of the parabiotic partner, and a binomial variable for the presence of each plant genus on the ant gardens. We allowed interactions for each of these variables with the climate PC1, because canopy cover or species distributions might be influenced by the climate. Both models were reduced in a stepwise manner until the AIC was lowest.

### 2.10 Statistical analyses—comparing data sets

To analyze associations between chemical profiles, genetic distance, and geographical distance, we performed Mantel tests based on Pearson correlation with 9,999 permutations. As measure for chemical distance (CHCs and polar substances separately), we used Bray–Curtis dissimilarities (command *vegdist*, package *vegan*, Oksanen et al., 2016). For each of the two haplotype pairs, Tamura–Nei (Tamura & Nei, 1993) pairwise genetic distances were calculated with MEGA7 based on the COI sequences. Geographical distances were measured as Euclidean distances between the GPS coordinates. All tests were done using R v. 3.5.0.

## 3 RESULTS

### 3.1 CHC differences between cryptic species

As described earlier (Emery & Tsutsui, 2013; Menzel et al., 2014), we found two clearly distinct chemotypes in both *Crematogaster levior* and *Camponotus femoratus*.

For *Crematogaster* (Figure 1c), the chemical networks yielded two large clusters in *Cr. levior* A (cluster XII and VII) and one large cluster in *Cr. levior* B (cluster V). The profiles of *Cr. levior* A seemed more variable as we found 13 different chemical types (with two singletons), compared with only 8 in *Cr. levior* B (with one singleton). *Crematogaster* A and B were clearly separated in the network. However, there was one exception, with the colony forming the singleton type XVIII showing characteristics of both chemotypes. In the network, it was closer connected to *Cr. levior* A, but clearly clustered with chemotype B in an NMDS ordination (Figure S1). This colony had the same COI haplotype as other B colonies.

**Figure 2:**
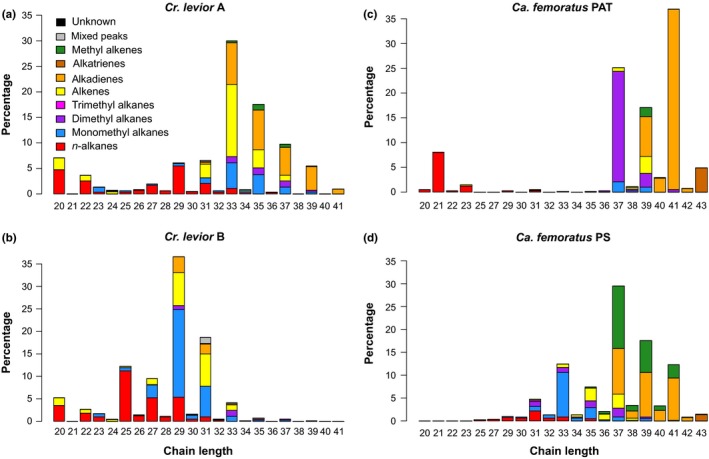
Differences between CHC profiles of the cryptic *Crematogaster levior* and *Camponotus femoratus* species. Plots show the mean distribution of different substance classes per chain length for all colonies of the respective species

The profile of *Cr. levior* A (*n* = 174) was dominated by several alkadienes of odd chain length ranging from C29 to C41 (total abundance: 27.44 ± 6.24%; Figure S3A). In contrast, the main peak in *Cr. levior* B (*n* = 148) was a mixture of 13‐ and 15‐methyl nonacosane (17.91 ± 7.73%; Figure S3B). The CHCs of both cryptic *Crematogaster* species were vastly different with substances most common in A (substances > 5% abundance: 30.24 ± 10.50%) being rare in B (6.36 ± 1.99%) and vice versa for substances most common in B (in B: 40.23 ± 9.51%; in A: 8.62 ± 3.37%). In comparison, the profile of *Cr. levior* A had more alkadienes (PERMANOVA: pseudo‐F_1_ = 137.98, *p* = .001), alkenes (pseudo‐F_1_ = 73.09, *p* = .001), dimethyl alkanes (pseudo‐*F*_1_ = 57.33, *p* = .001), and methyl‐branched alkenes (pseudo‐*F*_1_ = 155.24, *p* = .001; Figure 2a), while *Cr. levior* B had much higher proportions of monomethyl alkanes (pseudo‐*F*_1_ = 637.39, *p* = .001) and *n*‐alkanes (pseudo‐*F*_1_ = 191.56, *p* = .001; Figure 2b).

The two cryptic *Ca. femoratus* species were obviously distinct without any exceptions. *Ca. femoratus* PAT colonies were mostly assigned to a single cluster (cluster I) and few colonies to a second one (cluster II). In comparison, PS colonies were distributed among three chemical types (clusters III, IV, and V; Figure 1d).

In *Ca. femoratus* PAT (*n* = 195), the CHC profile was dominated by 13,23‐dimethyl heptatriacontane (22.47 ± 10.21%) and several different C41 alkadienes (13.24 ± 4.42% and 11.61 ± 3.42% for the two most abundant ones; Figure S3C). In *Ca. femoratus* PS (*n* = 107), the most abundant substance was a 13‐methyl heptatriacontene (13.49 ± 4.01%) followed by 13‐ and 15‐methyl tritriacontane (9.60 ± 2.75%; Figure S3D). The profiles of the cryptic *Camponotus* species differed strongly with the most common CHCs of *Ca. femoratus* PAT (substances > 5% abundance: 62.75 ± 7.24%) being less common in PS (9.67 ± 2.60%) and the other way around although less pronounced (in PS: 50.55 ± 8.23%; in PAT: 19.19 ± 5.19%). The PAT colonies had higher proportions of dimethyl alkanes (PERMANOVA: pseudo‐*F*_1_ = 629.70, *p* = .001), alkadienes (pseudo‐*F*_1_ = 202.82, *p* = .001), and *n*‐alkanes (pseudo‐*F*_1_ = 16.87, *p* = .001, Figure 2c), while the PS ones had more monomethyl alkanes (pseudo‐*F*_1_ = 1,205.50, *p* = .001), methyl‐branched alkenes (pseudo‐*F*_1_ = 1,013.00, *p* = .001), and alkenes (pseudo‐*F*_1_ = 105.53, *p* = .001; Figure 2d).

### 3.2 Differentiation by polar metabolites

**Figure 3:**
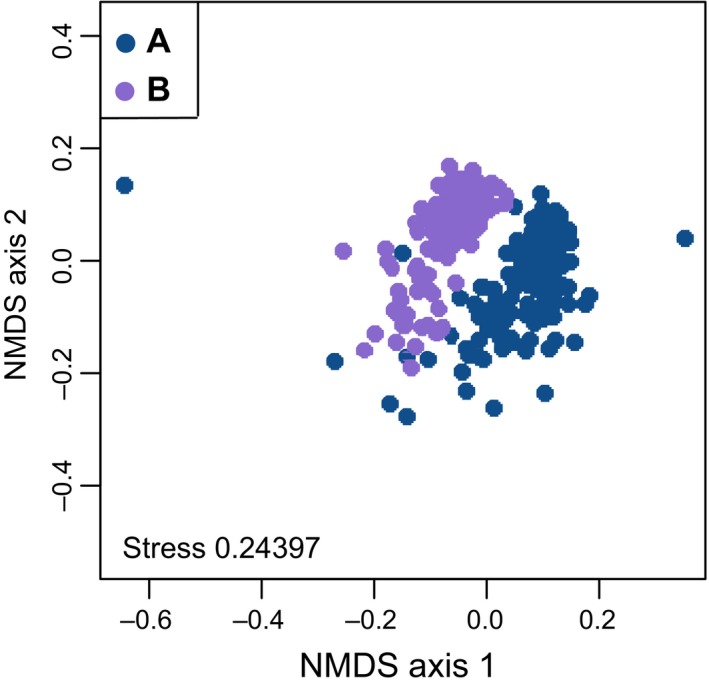
Differences in polar secondary metabolites of *Cr. levior*. NMDS ordination of the polar secondary metabolites produced by *Cr. levior*. Each dot represents the polar compound profile of one colony of *Cr. levior*

In 254 out of 322 *Cr. levior* colonies, we found a total of 60 different polar compounds on the cuticle. In the remaining extracts, polar substances were either not detected or had too low concentrations for reliable quantification. Similar to the CHCs, the colonies could be differentiated into two different clusters (Figure 3; Figure S4A,D). CHC chemotypes could be correctly identified based on polar chemistry using a random forest algorithm which had a 1.18% OOB estimate of error rate. All 138 samples from A and 113 of 116 samples of B (error rate of 0.026%) were classified correctly.

The most common substances in *Cr. levior* A had abundances of 8.55 ± 8.12% (retention time 24.10, Figure S4B), 8.74 ± 6.70% (RT 24.62), and 19.19 ± 10.32% (RT 26.24; Figure S4C), respectively, but lower abundances in B (3.45 ± 3.11%; 0.86 ± 1.07%; 3.82 ± 2.58%). In *Cr. levior* B, most abundant substances had proportions of 17.26 ± 10.81% (RT 20.20, Figure S4E), 13.55 ± 4.40% (RT 20.30, Figure S4F), and 5.45 ± 6.35% (RT 20.90), which were only 1.14 ± 1.13%, 3.35 ± 1.58%, and 0.44 ± 0.67%, respectively, in A (all retention times given refer to the Zebron Inferno ZB5‐MS capillary column). Using HR‐MS, the sum formulae of the major polar substances were derived as C_24_H_36_O_4_ (polar substance at retention time 20.20, Figure S4E), C_24_H_38_O_4_ (RT 20.30, Figure S4F), C_24_H_36_O_4_ (RT 20.90), C_26_H_38_O_4_ (RT 24.10, Figure S4B), C_26_H_40_O_4_ (RT 24.62), and C_28_H_44_O_4_ (RT 26.24, Figure S4C). The results showed a series of closely related compounds characterized by C24 to C28 carbon atoms containing four oxygen atoms, differing in the number of double bonds or rings from 6 to 8. In most cases, there was a pair of compounds showing the same number of carbons only differing in the number of double bonds/rings. This pairwise difference is also reflected in two series of fragment ions of *m*/*z* 237, 224, 209, and *m*/*z* 235, 222, 207, respectively, indicating an additional double bond isomer. However, to gain more insight into the underlying structures, higher quantities at higher purities are needed for NMR analysis.

### 3.3 Morphology

**Figure 4:**
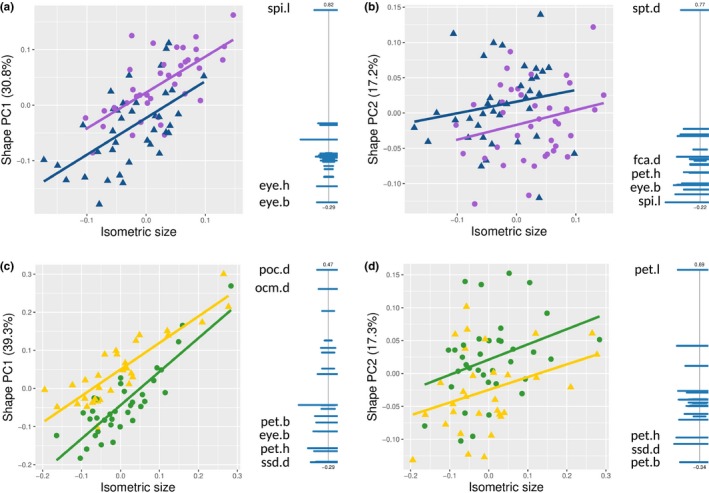
Morphological differentiation of the cryptic species of both ant genera. (a–d) Scatter plots depicting morphological differences of *Cr. levior* (a,b) and *Ca. femoratus* (c,d) and PCA ratio spectra. We plotted the first and second axis of a shape PCA (a, c and b, d, respectively) against isometric size. Each dot represents one individual of independent colonies. Symbols and colors correspond to cryptic species as follows: *Cr. levior*: blue triangle = A, purple dot = B; *Ca. femoratus*: yellow triangle = PAT, and green dot = PS. To the right of the scatterplots, the ratio spectrum of the shape PC is shown. Up to four of the most relevant variables for calculating body ratios are indicated the ends of the spectra using the variable codes (see Table S1). Bars indicate the 68% confidence intervals based on 1,000 bootstrap replicates (bars trimmed on right hand side due to the arrangement of figures)

In shape, the two cryptic species of *Cr. levior* were largely overlapping. Nevertheless, the shape significantly differed between them (MANOVA based on shape PCA: *F*_1_ = 18.07, *p* < .001) but not between sampling locations (*F*_11_ = 0.79, *p* = .73). *Cr. levior* A and B differed in shape PC1 (*F*_1_ = 30.37, *p* < .001; Figure 4a) but only insignificantly in shape PC2 (*F*_1_ = 3.18, *p* = .079; Figure 4b). Shape PC1 was best described by the ratio between spine length and eye width (Figure 4a), while shape PC2 was largely explained by the maximal distance between the spines (Figure 4b). Moreover, *Cr. levior* B was larger than A (Welch *t*‐test: *t*_74.94_ = −3.61, *p* < .001; Figure 4a,b).

The morphological traits of *Ca. femoratus* largely overlapped between cryptic species as well, despite significant differences (MANOVA: *F*_1_ = 16.67, *p* < .001). Again, we found no effect of sampling location (*F*_11_ = 0.43, *p* = .16). While we detected differences in body shape (shape PC1: *F*_1_ = 17.08, *p* = .001, Figure 3c; shape PC2: *F*_1_ = 10.04, *p* = .003, Figure 4d), the cryptic species did not differ in isometric size (t_57_ = −0.41, *p* = .68; Figure 4c,d). While the first shape PC was characterized by multiple traits on different body parts (Figure 4c), shape PC2 was mainly explained by the ratio between petiole length to petiole width (Figure 4d).

### 3.4 Genotyping results and population structure

#### 3.4.1 COI—parsimony networks and phylogeny

The TCS networks of the COI sequences show two distinct genotype clusters for both *Cr. levior* (Figure 1e) and *Ca. femoratus* (Figure 1f) with a 1:1 association of genotype to chemotype. The *Cr. levior* group that corresponds to A consisted of a single haplotype only. *Cr. levior* B showed more genetic variation with five haplotypes. The separation between both species was based on 16 SNPs (single nucleotide polymorphisms), indicating divergent clades. In *Ca. femoratus*, the resulting haplotype networks were more diverse. Both *Ca. femoratus* PS and PAT consisted of eight distinct groups. Here, the cryptic species were separated by two SNPs.

The phylogenies showed a similar pattern. In *Cr. levior*, the separation between cryptic species was strongly supported with a posterior probability of 1 (Figure S5). In *Ca. femoratus*, the separation was not as clear, based solely on COI with a posterior probability of .61 and two subgroups per cryptic species (Figure S6).

#### 3.4.2 COI—Population genetic structure

As measure for population differentiation, we calculated pairwise *F*_ST_ values separately for all four cryptic species, between all sampled sites. In *Cr. levior* A, results are not shown due to a lack of population differentiation (*F*_ST_ = 0 in all population comparisons). For *Cr. levior* B (Table 2), only few populations were genetically different with significant differentiation found between Kourou & Les Nouragues (*F*_ST_ = 0.308, *p* = .036), Kourou & Saint‐Laurent (*F*_ST_ = 0.531, *p* = .045), and Saint‐Laurent & Les Nouragues (*F*_ST_ = 0.127, *p* = .045). In *Ca. femoratus*, we found greater differentiation between populations compared with *Cr. levior*, with six occurrences of fixed differences (*F*_ST_ = 1). In 42% of all pairwise comparisons, populations were significantly different in PS (Table 3), and 29% of all comparisons in PAT yielded significant differences (Table 4). We furthermore tested for potential selection using Tajima's D statistic (Table S4). Results for *Cr. levior* A are again not shown due to a lack of genetic differences. In *Cr. levior* B, Tajima's D was not significant in any population. In *Ca. femoratus* PS, Tajima's D was significantly smaller than zero in the Saint‐Laurent population (TD = −1.513, *p* = .033) only. In *Ca. femoratus* PAT, Tajima's D was significantly smaller than zero in the populations of Paracou (TD = −2.072, *p* = .003), Les Nouragues (TD = −2.107, *p* = .002), and Saint‐Laurent (TD = −1.486, *p* = .04).

**Table 2:** Population pairwise *F*_ST_ between 10 populations of *Cr. levior* B, based on the COI locus

	AP	PAR	PS	LN	PAT	CAY	CA	KO	SI	SL
AP	–									
PAR	−.006	–								
PS	.000	−.130	–							
LN	.108	.112	.010	–						
PAT	−.012	−.006	−.117	.070	–					
CAY	.000	−.096	.000	.037	−.085	–				
CA	.000	−.031	.000	.088	−.030	.000	–			
KO	.462	.203	.195	**.308**	−.009	.250	.392	–		
SI	.034	.007	−.116	.104	−.025	−.078	.000	.253	–	
SL	.000	.017	.000	**.127**	.003	.000	.000	**.532**	.068	–

Note

Bold characters indicate statistical significance (*p* < .05) based on a permutation test.

**Table 3:** Population pairwise *F*_ST_ between nine populations of *Ca. femoratus* PS, based on the COI locus

	AP	PAR	PS	LN	RE	MT	KO	SI	SL
AP	–								
PAR	−.037	–							
PS	.156	**.092**	–						
LN	**1.000**	.778	**.796**	–					
RE	**1.000**	**.787**	**.811**	.000	–				
MT	1.000	**.778**	**.796**	.000	.000	–			
KO	.189	.001	−.135	.817	**.847**	.817	–		
SI	.000	−.054	.120	**1.000**	**1.000**	**1.000**	.126	–	
SL	.014	.009	.016	**.892**	**.897**	**.892**	−.079	−.008	–

Note

Bold characters indicate statistical significance (*p* < .05) based on a permutation test.

**Table 4:** Population pairwise *F*_ST_ between 12 populations of *Ca. femoratus* PAT, based on the COI locus

	AP	PAR	PS	LN	RE	PAT	CAY	MT	CA	KO	SI	SL
AP	–											
PAR	**.716**	–										
PS	.500	−.084	–									
LN	−.153	**.763**	**.637**	–								
RE	.248	**.205**	−.200	**.437**	–							
PAT	−.032	**.545**	**.273**	**.065**	**.130**	–						
CAY	.250	.173	−.333	**.451**	−.209	**.076**	–					
MT	.000	.694	.368	−.277	.164	−.133	.111	–				
CA	−.034	**.521**	.202	**.080**	.074	−.034	.010	−.144	–			
KO	.000	**.732**	.579	−.099	.296	.013	.333	.000	.017	–		
SI	.516	.001	−.273	**.648**	−.020	**.362**	−.108	.464	.307	**.551**	–	
SL	−.167	**.618**	.325	−.051	.141	−.063	.101	−.313	−.083	−.098	**.400**	–

Note

Bold characters indicate statistical significance (*p* < .05) based on a permutation test.

#### 3.4.3 *Camponotus* nuclear markers—parsimony networks and phylogeny

**Figure 5:**
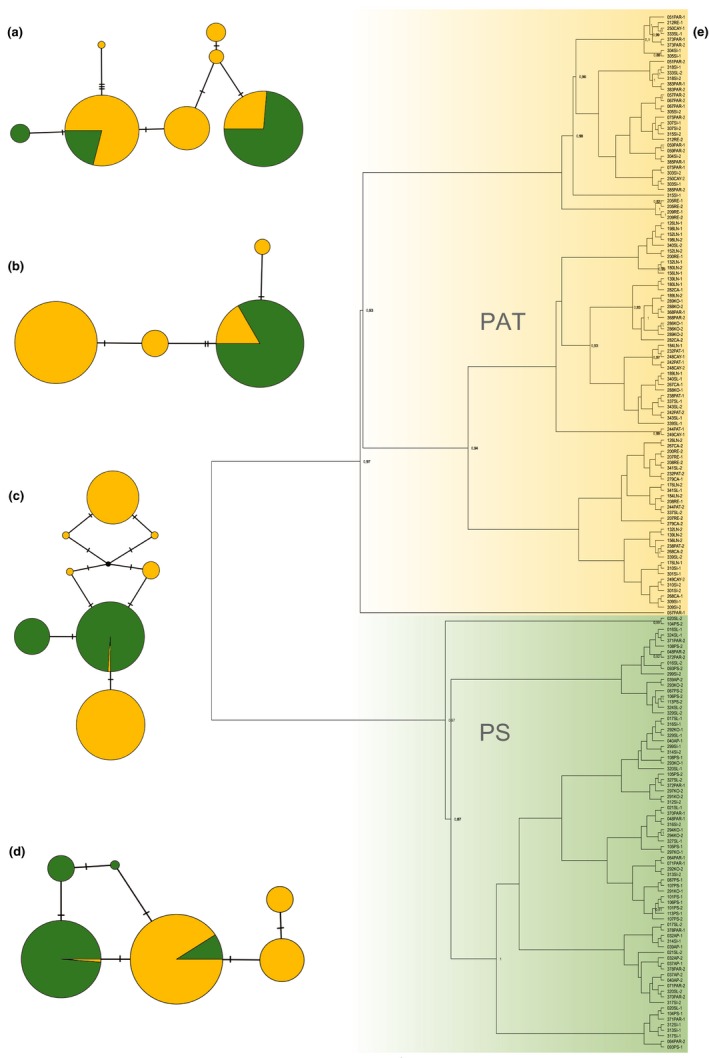
Genetic differentiation of cryptic *Ca. femoratus* species. (a–d) TCS Haplotype networks of four nuclear markers of *Ca. femoratus*. (a) ant.1401FR, (b) ant.1FR, (c) ant.1087FR, and (d) ant.389FR. Green color indicates *Ca. femoratus* PS, and yellow color indicates *Ca. femoratus* PAT, respectively. Haplotypes are shown as circles, with size depending on the number of included colonies. A number of SNPs (single nucleotide polymorphisms) between the haplotypes are shown as hatch marks. (e) Phylogenetic tree based on all four nuclear markers and mitochondrial COI for *Ca. femoratus*. Posterior probabilities >.8 are displayed. Yellow color corresponds to chemotype *Ca. femoratus* PAT, and green indicates *Ca. femoratus* PS. Only individuals with all five loci sequenced were included (*N* = 93)

As for COI sequences, we constructed TCS parsimony networks based on four additional nuclear markers (Figure 5a–d; we sequenced additional nuclear loci for *Camponotus* only, since a population genomic study is on the way for *Crematogaster*). In contrast to the network based on COI mitochondrial sequences, the networks of nuclear markers showed less clear separation of cryptic species (Figure 5a–d). In contrast, a phylogenetic tree based on all five sequenced markers (Figure 5e) clearly separated *Ca. femoratus* PAT and PS into two clades. Also, the STRUCTURE analysis showed that all individuals could be assigned to one of the two chemotypes (Figure S7).

### 3.5 Partner preference and environmental association of cryptic species

There was no indication for a preferred association between either cryptic *Cr. levior* or *Ca. femoratus* species (Pearson's chi‐squared test: $\chi_{1}^{2}$ = 1.76, *p* = .18). *Cr. levior* A nested with *Ca. femoratus* PAT in 100 and with PS in 65 cases, while *Cr. levior* B cohabited 96 times with *Camponotus* PAT and 44 times with PS.

The distribution of cryptic *Crematogaster* species was independent of PC1, that is, precipitation and temperature (binomial GLM: *N* = 292, $\chi_{1}^{2}$ = 1.12, *p* = .29), indicating sympatric occurrence of the cryptic species which is also visible when looking at their distribution across the complete sampling range (Figure 1a). Neither canopy cover nor the presence of any plant influenced the probability of species membership (A vs. B) in *Crematogaster* (all *p* > .2). However, species identity was influenced by an interaction of climate and *Camponotus* partner ($\chi_{1}^{2}$ = 5.97, *p* = .015). *Ca. femoratus* PS was less common in areas with high annual precipitation and lower annual mean temperature (i.e., the eastern part of French Guiana), while *Ca. femoratus* PAT was present across the whole sampling area (binomial GLM: *N* = 279, climate PC1: $\chi_{1}^{2}$ = 111.91, *p* < .001; Figure 1b). None of the other factors tested influenced the probability of the species' presence (all *p* > .15). However, there was a weak interaction between climate PC1 and *Crematogaster* partner ($\chi_{1}^{2}$ = 5.06, *p* = .025), indicating slightly differing partner availability depending on climate.

### 3.6 Connecting chemical profiles, genetic background, and geographic distance

The CHC distances in *Cr. levior* A slightly increased with geographic distance (Mantel test: *r* = .066, *p* = .011). However, this was not true for *Cr. levior* B (*r* = .044, *p* = .084). In *Camponotus*, CHC distances increased with geographical distances for PS (*r* = .182, *p* < .001), but not for PAT (*r* = .040, *p* = .15). The Bray–Curtis dissimilarities of CHCs and polar compounds of *Crematogaster* were highly correlated (*N* = 253, *r* = .42, *p* < .001), further indicating that the polar differentiation exactly matches the CHC differentiation. However, within each cryptic species, CHC distance and distance in polar compounds were not correlated (*Cr. levior* A: *r* = .04, *p* = .15; *Cr. levior* B: *r* = .02, *p* = .34).

Mantel tests between pairwise Tamura–Nei distances and geographic distances revealed no isolation‐by‐distance pattern for *Cr. levior* B (*r* = −.065, *p* = .968), but for *Ca. femoratus* PS (*r* = .38, *p* < .001) and—albeit only weakly—*Ca. femoratus* PAT (*r* = .09, *p* = .038). *Cr. levior* A consisted of only one haplotype without any variation at the COI locus, which is why this analysis was not possible here.

In *Cr. levior* B, colonies that were genetically more distant also had more dissimilar CHC profiles (*r* = .15, *p* = .021). However, such an association was detectable neither within *Ca. femoratus* PAT (*r* = .05, *p* = .15) nor within *Ca. femoratus* PS (*r* = .03, *p* = .29).

## 4 DISCUSSION

This study investigated the parabiotic ant species *Cr. levior* and *Ca. femoratus* whose shared nests (so‐called ant gardens) are abundant in the neotropics (Davidson, 1988). Both previously identified species occur in two distinct CHC chemotypes, which are morphologically highly similar. We show that within *Cr. levior* and within *Ca. femoratus*, these chemotypes form two distinct units that can be classified as cryptic species. This is supported by multiple lines of evidence, all of which show conclusive results. First, the cuticular hydrocarbon analysis shows that both formerly classified species split into two clearly distinguishable chemotypes across our sampling range without intermediate profiles. For *Cr. levior*, we additionally show a clear separation in polar metabolites. Secondly, we morphometrically analyzed the different species. Although there is a large overlap in traits between groups, we found slight but significant differences in body shape between the two cryptic *Camponotus* and between the two cryptic *Crematogaster* species. Moreover, *Cr. levior* B is slightly larger than *Cr. levior* A. Lastly, we barcoded all sampled colonies and found a 1:1 association between the previously assigned CHC chemotypes and newly assigned genotypes. Phylogenies based on COI perfectly split *Cr. levior* into two clusters. The same holds true for *Ca. femoratus* based on COI and four additional nuclear markers, where again two distinct clusters are found. These results support our initial hypothesis that apparent CHC diversity is in fact a sign of distinct genetic lineages, that is, cryptic species (in the sense of De Queiroz, 2007). In the following sections, we first discuss the distribution and ecological niches of the cryptic species, then their population structures and possible scenarios explaining those, and lastly, the putative role of the vastly different cuticular hydrocarbon profiles during or after the speciation process.

Previous studies that looked at the distribution of cryptic species mostly found evidence for the competitive exclusion principle (García‐Robledo, Kuprewicz, Staines, Erwin, & Kress, 2015; Leavitt, Starrett, Westphal, & Hedin, 2015; Vodă, Dapporto, Dincă, & Vila, 2015). In fig wasps for example, morphologically similar species are less likely to occur in sympatry than morphologically dissimilar sister species (Darwell & Cook, 2017). Interestingly, in our case, the two *Crematogaster* and *Camponotus* sister species co‐occur across the whole sampling range with only one case of niche differentiation within the factors tested here. *Camponotus femoratus* PS is more common in the drier, western half of the country, while PAT was more frequently found in the wetter and slightly cooler east of the country. The high proportions of alkadienes in the CHC profile of *Ca. femoratus* PAT are in line with this climatic difference. This corroborates other studies in which alkadienes were found to be present more frequently and in higher percentages (only in interaction with cooler temperature) in multiple different species from high precipitation areas (Menzel, Blaimer, & Schmitt, 2017; van Wilgenburg, Symonds, & Elgar, 2011). In contrast, the two *Crematogaster* species occur in similar frequencies across the whole sampling range with no obvious signs for niche differentiation in the parameters we tested. However, other ecological parameters such as dietary differences or niche partitioning concerning the time of foraging activity or mating flights may still be of importance. Alternatively, *Cr. levior* A and B may represent ecologically neutral species (Adler, HilleRisLambers, & Levine, 2007; Bell, 2017; Hubbell, 2001). In this scenario, diverse communities of functionally equivalent species coexist due to neutral dynamics (Hubbell, 2005). We furthermore found no preferential association of either *Crematogaster* species for any of the two *Camponotus* species or vice versa, rendering cospeciation a more unlikely scenario. The lack in preference may not be too surprising given the distribution of the species. While the two *Crematogaster* species occur in similar frequencies throughout the sampling range, the two *Camponotus* species show the above‐mentioned east–west gradient. The choice of the mutualistic partner might therefore be a question of availability rather than preference.

Population structure and haplotype diversity differed strongly between species. It was most extreme, with only a single haplotype and no population differentiation in *Cr. levior* A between all 12 sampled populations. We found five different haplotypes in *Cr. levior* B and eight in both *Ca. femoratus* species. In *Cr. levior* B, population structure was very weak and there was no sign for isolation by distance. This result is surprising insofar, as other studies on the genus *Crematogaster* usually show strong geographical or ecological structure (Boyle, Martins, Musili, & Pierce, 2018; Türke, Fiala, Linsenmair, & Feldhaar, 2010). In *Ca. femoratus* PS and PAT, respectively, the COI locus and two nuclear markers showed clear signs for isolation by distance. Tajima's *D* analysis furthermore showed signs for sudden population expansions in several of the observed populations of *Ca. femoratus* PS and PAT. Genetic differences between the two *Camponotus* species were generally low and only a small part of the nuclear markers we tested were variable between species. Furthermore, the previously assigned CHC chemotypes did not perfectly match the haplotypes of any of the nuclear loci, which may be due to incomplete lineage sorting, a possible sign of recent speciation between *Ca. femoratus* PS and *Ca. femoratus* PAT.

The lack of any population differentiation in *Cr. levior* A, with only a single COI haplotype in all sampled populations, could be explained by two different scenarios. The first is a strong bottleneck event coupled with a recent population expansion. A second explanation could be a selective sweep in haplotype A together with a population expansion. In insects, this is often found in the context of an infection with the endosymbiont *Wolbachia* that can manipulate its hosts reproduction (through e.g., mate‐discrimination, cytoplasmic incompatibilities; Hoffmann, Turelli, & Simmons, 1986; Schuler et al., 2016). However, the same signatures can be found after the spread of a beneficial mutation within a population, that will lead to reduced heterozygosity around the selected locus (Schlenke & Begun, 2004). While we found only weak genetic differences between the cryptic *Camponotus* species, chemical differences were pronounced. Also, *Crematogaster* showed unusually high interspecific differences in their chemical profile, which has previously been discussed as a mechanism to reinforce species divergence (Menzel, Schmitt, & Blaimer, 2017). The overlap in CHC composition between the two species of each genus was low, with peaks that were abundant in one species being low or absent in the other (see Section 3.1). This means that the CHC profiles differ much more than one would expect between sister species sharing similar abiotic and biotic niches (Menzel, Schmitt, et al., 2017). Especially compared with other traits, for example, morphology or behavior, chemical trait differences seem to be higher and less phylogenetically conserved (Blomberg, Garland, & Ives, 2003; Kamilar & Cooper, 2013). Chemical distance and genetic distance were correlated in *Cr. levior* B—but not in A, or any of the cryptic *Ca. femoratus* species. Interestingly, in *Cr. levior* A, in which we only found a single COI haplotype, the chemical diversity was very large compared with the uniformity we observed in the COI locus. Taken together, this in our opinion suggests that the CHC divergence may have played a role in species divergence—either during or after speciation. The main role of cuticular hydrocarbons is to serve as desiccation barrier but, especially in social insects, additionally play a role in communication and as mating cues (Thomas & Simmons, 2008). They therefore have been discussed as possible “magic traits,” that is, traits that affect both ecological adaptation and mate signaling (Chung & Carroll, 2015; Smadja & Butlin, 2009), which can be mediated by a single gene only (Chung et al., 2014). Changes in such traits will often lead to assortative mating and ultimately to speciation (Chung & Carroll, 2015). In *Timema* stick insects, speciation events were generally associated with a divergence in CHC profiles; however, it remained unclear whether speciation followed CHC divergence or whether CHC profiles diverged due to selection during the evolution of reproductive isolation (Schwander et al., 2013). The same holds true for both cryptic species pairs in *Crematogaster* and *Camponotus*. The surprisingly high chemical divergence, combined with low genetic diversity (at least in *Camponotus*), might be indicative for a role of CHCs in species divergence. But it remains to be elucidated whether CHCs played a role in the speciation event itself by inducing assortative mating, by reinforcing sexual selection after the speciation event, or by niche partitioning, that is, adaptation to a yet unknown factor.

## 5 CONCLUSION

We could conclusively show that both *Crematogaster levior* and *Camponotus femoratus* split into two morphologically nearly indistinguishable cryptic species. It remains unclear how speciation took place in the two genera, but the strong separation in cuticular hydrocarbon profiles suggests that they are involved in mediating species divergence. Since *Crematogaster levior* and *Camponotus femoratus* are only found in mutualistic associations, we were rather surprised to find no partner preferences as indication for cospeciation in this mutualistic complex. Moreover, the highly different population structures between and within genera point to a rather loose relationship among the mutualists, whereas similar population structures would be expected if there was a strict partner specialization. Future studies should investigate partner choice and recognition, the evolution of the distinct chemotypes, the phylogeography of the species, as well as genome wide patterns of selection to shed further light on this highly interesting association and its players. This will help to deepen our knowledge on the effect of mutualistic interactions on species divergence.

## CONFLICT OF INTEREST

None declared.

## AUTHOR CONTRIBUTIONS

TS, BF, and FM: conceived the study. JH, PPS, JO, BF, and FM: collected the specimens and field data. PPS, JS, TB, TS, and FM: did the chemical analyses and respective data analyses. JS and HB: did the morphological measurements and corresponding statistical analysis. JH, HW, and BF: performed sequencing and genetic analyses. JH, PPS, BF, and FM: wrote the first version of the manuscript. HB and TB: added to the methods and results sections. All authors contributed to writing this version and approved the submission.

## Data Availability

Sequence alignments, morphological data, and chemical datasets are available on Dryad (DOI: https://doi.org/10.5061/dryad.9r106tn).

## References

[ece35464-bib-0001] Adler, P. B. , HilleRisLambers, J. , & Levine, J. M. (2007). A niche for neutrality. Ecology Letters, 10, 95–104. 10.1111/j.1461-0248.2006.00996.x 17257097

[ece35464-bib-0002] Aitchison, J. (1982). The statistical analysis of compositional data. Journal of the Royal Statistical Society: Series B (Methodological), 44, 139–177. 10.1111/j.2517-6161.1982.tb01195.x

[ece35464-bib-0003] Andersson, M. (1982). Sexual selection, natural selection and quality advertisement. Biological Journal of the Linnean Society, 17, 375–393. 10.1111/j.1095-8312.1982.tb02028.x

[ece35464-bib-0004] Bartlett, J. W. , & Frost, C. (2008). Reliability, repeatability and reproducibility: Analysis of measurement errors in continuous variables. Ultrasound in Obstetrics and Gynecology, 31, 466–475. 10.1002/uog.5256 18306169

[ece35464-bib-0005] Baur, H. , Kranz‐Baltensperger, Y. , Cruaud, A. , Rasplus, J. Y. , Timokhov, A. V. , & Gokhman, V. E. (2014). Morphometric analysis and taxonomic revision of *Anisopteromalus* Ruschka (Hymenoptera: Chalcidoidea: Pteromalidae) – An integrative approach. Systematic Entomology, 39, 691–709. 26074661 10.1111/syen.12081 PMC4459240

[ece35464-bib-0006] Baur, H. , & Leuenberger, C. (2011). Analysis of ratios in multivariate morphometry. Systematic Biology, 60, 813–825. 10.1093/sysbio/syr061 21828084 PMC3193766

[ece35464-bib-0007] Bell, G. (2017). The distribution of abundance in neutral communities. The American Naturalist, 155, 606 10.2307/3078983 10777433

[ece35464-bib-0008] Bickford, D. , Lohman, D. J. , Sodhi, N. S. , Ng, P. K. L. , Meier, R. , Winker, K. , … Das, I. (2007). Cryptic species as a window on diversity and conservation. Trends in Ecology & Evolution, 22, 148–155. 10.1016/j.tree.2006.11.004 17129636

[ece35464-bib-0009] Blomberg, S. P. , Garland, T. , & Ives, A. R. (2003). Testing for phylogenetic signal in comparative data: Behavioral traits are more labile. Evolution, 57, 717–745. 12778543 10.1111/j.0014-3820.2003.tb00285.x

[ece35464-bib-0010] Blomquist, G. J. (2010). Structure and analysis of insect hydrocarbons In Blomquist G. J., & Bagnères A.‐G. (Eds.), Insect hydrocarbons: Biology, biochemistry, and chemical ecology (pp. 19–34). New York, NY: Cambridge University Press.

[ece35464-bib-0011] Blomquist, G. J. , & Bagnères, A.‐G. (2010). Introduction: History and overview of insect hydrocarbons In Blomquist G. J., & Bagnères A.‐G. (Eds.), Insect hydrocarbons: Biology, biochemistry, and chemical ecology (pp. 3–18). New York, NY: Cambridge University Press.

[ece35464-bib-0012] Bolaños, L. M. , Rosenblueth, M. , Manrique de Lara, A. , Migueles‐Lozano, A. , Gil‐Aguillón, C. , Mateo‐Estrada, V. , … Martínez‐Romero, E. (2019). Cophylogenetic analysis suggests cospeciation between the Scorpion *Mycoplasma* Clade symbionts and their hosts. PLoS ONE, 14, e0209588 10.1371/journal.pone.0209588 30625167 PMC6326461

[ece35464-bib-0013] Bouckaert, R. , Heled, J. , Kühnert, D. , Vaughan, T. , Wu, C.‐H. , Xie, D. , … Drummond, A. J. (2014). BEAST 2: A Software Platform for Bayesian Evolutionary Analysis. PLoS Computational Biology, 10, e1003537 10.1371/journal.pcbi.1003537 24722319 PMC3985171

[ece35464-bib-0014] Boyle, J. H. , Martins, D. , Musili, P. M. , & Pierce, N. E. (2018). Population genomics and demographic sampling of the ant‐plant *Vachellia drepanolobium* and its symbiotic ants from sites across its range in East Africa. Frontiers in Ecology and Evolution, 7, 206 10.3389/fevo.2019.00206

[ece35464-bib-0015] Brückner, A. , & Heethoff, M. (2017). A chemo‐ecologists' practical guide to compositional data analysis. Chemoecology, 27, 33–46. 10.1007/s00049-016-0227-8

[ece35464-bib-0016] Carlson, D. A. , Bernier, U. R. , & Sutton, B. D. (1998). Elution patterns from capillary GC for methyl‐branched alkanes. Journal of Chemical Ecology, 24, 1845–1865.

[ece35464-bib-0017] Chomicki, G. , Ward, P. S. , & Renner, S. S. (2015). Macroevolutionary assembly of ant/plant symbioses: *Pseudomyrmex* ants and their ant‐housing plants in the Neotropics. Proceedings of the Royal Society B: Biological Sciences, 282, 20152200. 10.1098/rspb.2015.2200 PMC4685824 26582029

[ece35464-bib-0018] Chung, H. , & Carroll, S. B. (2015). Wax, sex and the origin of species: Dual roles of insect cuticular hydrocarbons in adaptation and mating. BioEssays, 37, 822–830. 10.1002/bies.201500014 25988392 PMC4683673

[ece35464-bib-0019] Chung, H. , Loehlin, D. W. , Dufour, H. D. , Vaccaro, K. , Millar, J. G. , & Carroll, S. B. (2014). A single gene affects both ecological divergence and mate choice in Drosophila. Science, 343(6175), 1148–1151. 24526311 10.1126/science.1249998

[ece35464-bib-0020] Cruaud, A. , Rønsted, N. , Chantarasuwan, B. , Chou, L. S. , Clement, W. L. , Couloux, A. , … Savolainen, V. (2012). An extreme case of plant – insect codiversification: Figs and fig‐pollinating wasps. Systematic Biology, 61, 1029–1047. 10.1093/sysbio/sys068 22848088 PMC3478567

[ece35464-bib-0021] Csösz, S. , Wagner, H. C. , Bozsó, M. , Seifert, B. , Arthofer, W. , Schlick‐Steiner, B. C. , … Pénzes, Z. (2014). *Tetramorium indocile* Santschi, 1927 stat. rev. is the proposed scientific name for *Tetramorium* sp. C sensu Schlick‐Steiner et al. (2006) based on combined molecular and morphological evidence (Hymenoptera: Formicidae). Zoologischer Anzeiger, 253, 469–481.

[ece35464-bib-0022] Darwell, C. T. , & Cook, J. M. (2017). Cryptic diversity in a fig wasp community — morphologically differentiated species are sympatric but cryptic species are parapatric. Molecular Ecology, 26, 937–950. 10.1111/mec.13985 28026893

[ece35464-bib-0023] Davidson, D. W. (1988). Ecological studies of Neotropical ant gardens. Ecology, 69, 1138–1152. 10.2307/1941268

[ece35464-bib-0024] De Queiroz, K. (2007). Species concepts and species delimitation. Systematic Biology, 56, 879–886. 10.1080/10635150701701083 18027281

[ece35464-bib-0025] de Vienne, D. M. , Refrégier, G. , López‐Villavicencio, M. , Tellier, A. , Hood, M. E. , & Giraud, T. (2013). Cospeciation vs host‐shift speciation: Methods for testing, evidence from natural associations and relation to coevolution. New Phytologist, 198, 347–385. 10.1111/nph.12150 23437795

[ece35464-bib-0026] Degnan, P. H. , Lazarus, A. B. , Brock, C. D. , & Wernegreen, J. J. (2004). Host – symbiont stability and fast evolutionary rates in an ant – Bacterium Association: Cospeciation of *Camponotus* species and their endosymbionts, *Candidatus* Blochmannia. Systematic Biology, 53, 95–110. 10.1080/10635150490264842 14965905

[ece35464-bib-0027] Dieckmann, U. , & Doebeli, M. (1999). On the origin of species by sympatric speciation. Nature, 400, 354–357. 10.1038/22521 10432112

[ece35464-bib-0028] Doebeli, M. , & Dieckmann, U. (2000). Evolutionary branching and sympatric speciation caused by different types of ecological interactions. The American Naturalist, 156, S77–S101. 10.1086/303417 29592583

[ece35464-bib-0029] Emery, V. J. , & Tsutsui, N. D. (2013). Recognition in a social symbiosis: Chemical phenotypes and nestmate recognition behaviors of Neotropical parabiotic ants. PLoS ONE, 8, e56492 10.1371/journal.pone.0056492 23451053 PMC3579830

[ece35464-bib-0030] Excoffier, L. , & Lischer, H. E. L. (2010). Arlequin suite ver 3.5: A new series of programs to perform population genetics analyses under Linux and Windows. Molecular Ecology Resources, 10, 564–567. 21565059 10.1111/j.1755-0998.2010.02847.x

[ece35464-bib-0031] García‐Robledo, C. , Kuprewicz, E. K. , Staines, C. L. , Erwin, T. L. , & Kress, W. J. (2015). Limited tolerance by insects to high temperatures across tropical elevational gradients and the implications of global warming for extinction. Proceedings of the National Academy of Sciences of the United States of America, 113, 680–685. 10.1073/pnas.1507681113 PMC4725502 26729867

[ece35464-bib-0032] Gause, G. F. (1932). Experimental studies on the struggle for existence I. Mixed population of two species of yeast. Journal of Experimental Biology, 9, 389–402.

[ece35464-bib-0033] Gebiola, M. , Monti, M. M. , Johnson, R. C. , Woolley, J. B. , Hunter, M. S. , Giorgini, M. , & Pedata, P. A. (2017). A revision of the *Encarsia pergandiella* species complex (Hymenoptera: Aphelinidae) shows cryptic diversity in parasitoids of whitefly pests. Systematic Entomology, 42, 31–59.

[ece35464-bib-0034] Grundt, H. H. , Kjølner, S. , Borgen, L. , Rieseberg, L. H. , & Brochmann, C. (2006). High biological species diversity in the arctic flora. Proceedings of the National Academy of Sciences of the United States of America, 103, 972–975. 10.1073/pnas.0510270103 16418291 PMC1348009

[ece35464-bib-0035] Guimarães, P. R. , Jordano, P. , & Thompson, J. N. (2011). Evolution and coevolution in mutualistic networks. Ecology Letters, 14, 877–885. 10.1111/j.1461-0248.2011.01649.x 21749596

[ece35464-bib-0036] Gustafson, K. D. , Kensinger, B. J. , Bolek, M. G. , & Luttbeg, B. (2014). Distinct snail (*Physa*) morphotypes from different habitats converge in shell shape and size under common garden conditions. Evolutionary Ecology Research, 16, 77–89.

[ece35464-bib-0037] Han, M. V. , & Zmasek, C. M. (2009). PhyloXML: XML for evolutionary biology and comparative genomics. BMC Bioinformatics, 10, 356 10.1186/1471-2105-10-356 19860910 PMC2774328

[ece35464-bib-0038] Hardin, G. (1960). The competitive exclusion principle. Science, 131, 1292–1297. 14399717 10.1126/science.131.3409.1292

[ece35464-bib-0039] Heethoff, M. , Laumann, M. , Weigmann, G. , & Raspotnig, G. (2011). Integrative taxonomy: Combining chemical, morphological and molecular data for delineation of the parthenogenetic *Trhypochthonius tectorum* complex (Acari, Oribatida, Trhypochthoniidae). Frontiers in Zoology, 8, 2. 21303503 10.1186/1742-9994-8-2 PMC3045314

[ece35464-bib-0040] Hoeksema, J. D. , & Bruna, E. M. (2000). Pursuing the big questions about interspecific mutualism: A review of theoretical approaches. Oecologia, 125, 321–330. 10.1007/s004420000496 28547326

[ece35464-bib-0041] Hoffmann, A. A. , Turelli, M. , & Simmons, G. M. (1986). Unidirectional incompatibility between populations of *Drosophila simulans* . Evolution, 40, 692–701. 28556160 10.1111/j.1558-5646.1986.tb00531.x

[ece35464-bib-0042] Hosokawa, T. , Kikuchi, Y. , Nikoh, N. , Shimada, M. , & Fukatsu, T. (2006). Strict Host‐Symbiont cospeciation and reductive genome evolution in insect gut bacteria. PLoS Biology, 4, e337 10.1371/journal.pbio.0040337 17032065 PMC1592312

[ece35464-bib-0043] Hubbell, S. P. (2001). The unified neutral theory of biodiversity and biogeography. Princeton, NJ: Princeton University Press.

[ece35464-bib-0044] Hubbell, S. P. (2005). Neutral theory in community ecology and the hypothesis of functional equivalence. Functional Ecology, 19, 166–172. 10.1111/j.0269-8463.2005.00965.x

[ece35464-bib-0045] Hudson, E. J. , & Price, T. D. (2014). Pervasive reinforcement and the role of sexual selection in biological speciation. Journal of Heredity, 105, 821–833. 10.1093/jhered/esu041 25149257

[ece35464-bib-0046] Janz, N. , Nyblom, K. , & Nylin, S. (2001). Evolutionary dynamics of host‐plant specialization: A case study of the Tribe *Nymohalini* . Evolution, 55, 783–796. 11392396 10.1554/0014-3820(2001)055[0783:edohps]2.0.co;2

[ece35464-bib-0047] Jousselin, E. , van Noort, S. , Berry, V. , Rasplus, J.‐Y. , Rønsted, N. , Erasmus, J. C. , & Greeff, J. M. (2008). One fig to bind them all: Host conservatism in a fig wasp community unraveled by cospeciation analyses among pollinating and nonpollinating fig wasps. Evolution, 62, 1777–1797. 10.1111/j.1558-5646.2008.00406.x 18419750

[ece35464-bib-0048] Kamilar, J. M. , & Cooper, N. (2013). Phylogenetic singal in primate behaviour, ecolog anf life history. Philosophical Transactions of the Royal Society of London. Series B, 368, 20120341. 23569289 10.1098/rstb.2012.0341 PMC3638444

[ece35464-bib-0049] Karger, D. N. , Conrad, O. , Böhner, J. , Kawohl, T. , Kreft, H. , Soria‐Auza, R. W. , … Kessler, M. (2017). Climatologies at high resolution for the earth's land surface areas. Scientific Data, 4, 170122 10.1038/sdata.2017.122 28872642 PMC5584396

[ece35464-bib-0050] Kawakita, A. , Takimura, A. , Terachi, T. , Sota, T. , & Kato, M. (2004). Cospeciation analysis of an obligate pollination mutualism: Have *Glochidon* trees (Euphorbiaceae) and pollinating *Epicephala* moths (Gracillaridae) diverified in parallel? Evolution, 58, 2201–2214. 15562685 10.1111/j.0014-3820.2004.tb01598.x

[ece35464-bib-0051] Klingenberg, C. P. (2016). Size, shape, and form: Concepts of allometry in geometric morphometrics. Development Genes and Evolution, 226, 113–137. 10.1007/s00427-016-0539-2 27038023 PMC4896994

[ece35464-bib-0052] Kumar, S. , Stecher, G. , Li, M. , Knyaz, C. , & Tamura, K. (2018). MEGA X: Molecular Evolutionary Genetics Analysis across computing platforms. Molecular Biology and Evolution, 35, 1547–1549. 10.1093/molbev/msy096 29722887 PMC5967553

[ece35464-bib-0053] Leavitt, D. H. , Starrett, J. , Westphal, M. F. , & Hedin, M. (2015). Multilocus sequence data reveal dozens of putative cryptic species in a radiation of endemic Californian mygalomorph spiders (Araneae, Mygalomorphae, Nemesiidae). Molecular Phylogenetics and Evolution, 91, 56–67. 10.1016/j.ympev.2015.05.016 26025426

[ece35464-bib-0054] Leigh, J. W. , & Bryant, D. (2015). POPART: Full‐feature software for haplotype network construction. Methods in Ecology and Evolution, 6, 1110–1116.

[ece35464-bib-0055] Liaw, A. , & Wiener, M. (2002). Classification and regression by randomForest. R News, 2, 18–22.

[ece35464-bib-0056] Martin, S. J. , Helanterä, H. , & Drijfhout, F. P. (2008). Evolution of species‐specific cuticular hydrocarbon patterns in *Formica* ants. Biological Journal of the Linnean Society, 95, 131–140. 10.1111/j.1095-8312.2008.01038.x

[ece35464-bib-0057] Menzel, F. , Blaimer, B. B. , & Schmitt, T. (2017). How do cuticular hydrocarbons evolve? Physiological constraints and climatic and biotic selection pressures act on a complex functional trait. Proceedings of the Royal Society B‐Biological Sciences, 284, 20161727 10.1098/rspb.2016.1727 PMC5360911 28298343

[ece35464-bib-0058] Menzel, F. , Linsenmair, K. E. , & Blüthgen, N. (2008). Selective interspecific tolerance in tropical *Crematogaster*‐*Camponotus* associations. Animal Behavior, 75, 837–846. 10.1016/j.anbehav.2007.07.005

[ece35464-bib-0059] Menzel, F. , Orivel, J. , Kaltenpoth, M. , & Schmitt, T. (2014). What makes you a potential partner? Insights from convergently evolved ant‐ant symbioses. Chemoecology, 24, 105–119. 10.1007/s00049-014-0149-2

[ece35464-bib-0060] Menzel, F. , Schmitt, T. , & Blaimer, B. B. (2017). The evolution of a complex trait: Cuticular hydrocarbons in ants evolve independent from phylogenetic constraints. Journal of Evolutionary Biology, 30, 1372–1385. 10.1111/jeb.13115 28485028

[ece35464-bib-0061] Montero‐Pau, J. , Gomez, A. , & Muñoz, J. (2008). Application of an inexpensive and high‐throughput genomic DNA extraction method for the molecular ecology of zooplanktonic diapausing eggs. Limnology and Oceanography: Methods, 6, 218–222. 10.4319/lom.2008.6.218

[ece35464-bib-0062] Nosil, P. (2012). Ecological speciation. Oxford, UK: Oxford University Press.

[ece35464-bib-0063] Oksanen, J. , Blanchet, F. G. , Friendly, M. , Kindt, R. , Legendre, P. , McGlinn, D. , … Wagner, H. (2016). vegan: Community Ecology Package. https://cran.r-project.org/web/packages/vegan/

[ece35464-bib-0064] Orivel, J. , Errard, C. , & Dejean, A. (1997). Ant gardens: Interspecific recognition in parabiotic ant species. Behavioral Ecology and Sociobiology, 40, 87–93. 10.1007/s002650050319

[ece35464-bib-0065] Paradis, E. (2010). Pegas: An R package for population genetics with an integrated‐modular approach. Bioinformatics, 26, 419–420. 10.1093/bioinformatics/btp696 20080509

[ece35464-bib-0066] Quek, S.‐P. , Davies, S. J. , Itino, T. , & Pierce, N. E. (2004). Codiversification in an ant‐plant mutualism: Stem texture and the evolution of host use in *Crematogaster* (Formicidae: Myrmicinae) Inhabitants of *Macaranga* (Euphorbiaceae). Evolution, 58, 554–570. 15119439

[ece35464-bib-0067] R Core Team (2018). R: A language and environment for statistical computing. Vienna, Austria: R Foundation for Statistical Computing.

[ece35464-bib-0068] Rambaut, A. , Drummond, A. J. , Xie, D. , Baele, G. , & Suchard, M. A. (2018). Posterior summarization in Bayesian Phylogenetics using Tracer 1.7. Systematic Biology, 67, 901–904. 10.1093/sysbio/syy032 29718447 PMC6101584

[ece35464-bib-0069] Ronquist, F. , Teslenko, M. , Van Der Mark, P. , Ayres, D. L. , Darling, A. , Höhna, S. , … Huelsenbeck, J. P. (2012). MrBayes 3.2: Efficient bayesian phylogenetic inference and model choice across a large model space. Systematic Biology, 61, 539–542. 22357727 10.1093/sysbio/sys029 PMC3329765

[ece35464-bib-0070] Schlenke, T. A. , & Begun, D. J. (2004). Strong selective sweep associated with a transposon insertion in *Drosophila simulans* . Proceedings of the National Academy of Sciences of the United States of America, 101, 1626–1631. 10.1073/pnas.0303793101 14745026 PMC341797

[ece35464-bib-0071] Schuler, H. , Köppler, K. , Daxböck‐Horvath, S. , Rasool, B. , Krumböck, S. , Schwarz, D. , … Riegler, M. (2016). The hitchhiker's guide to Europe: The infection dynamics of an ongoing Wolbachia invasion and mitochondrial selective sweep in *Rhagoletis cerasi* . Molecular Ecology, 25, 1595–1609. 26846713 10.1111/mec.13571 PMC4950298

[ece35464-bib-0072] Schultz, T. R. , Solomon, S. A. , Mueller, U. G. , Villesen, P. , Boomsma, J. J. , Adams, R. M. M. , & Norden, B. (2002). Cryptic speciation in the fungus‐growing ants *Cyphomyrmex longiscapus* Weber and *Cyphomyrmex muelleri* Schultz and Solomon, new species (Formicidae, Attini). Insectes Sociaux, 49, 331–343. 10.1007/PL00012657

[ece35464-bib-0073] Schwander, T. , Arbuthnott, D. , Gries, R. , Gries, G. , Nosil, P. , & Crespi, B. J. (2013). Hydrocarbon divergence and reproductive isolation in *Timema* stick insects. BMC Evolutionary Biology, 13, 151 10.1186/1471-2148-13-151 23855797 PMC3728149

[ece35464-bib-0074] Scriven, J. J. , Whitehorn, P. R. , Goulson, D. , & Tinsley, M. C. (2016). Niche partitioning in a sympatric cryptic species complex. Ecology and Evolution, 6, 1328–1339. 10.1002/ece3.1965 26848386 PMC4730923

[ece35464-bib-0075] Seifert, B. (2008). Removal of allometric variance improves species separation in multi‐character discriminant functions when species are strongly allometric and exposes diagnostic characters. Myrmecological News, 11, 91–105.

[ece35464-bib-0076] Servedio, M. R. , Van Doorn, G. S. , Kopp, M. , Frame, A. M. , & Nosil, P. (2011). Magic traits in speciation: “magic” but not rare? Trends in Ecology & Evolution, 26, 389–397. 21592615 10.1016/j.tree.2011.04.005

[ece35464-bib-0077] Smadja, C. , & Butlin, R. K. (2009). On the scent of speciation: The chemosensory system and its role in premating isolation. Heredity, 102, 77–97. 10.1038/hdy.2008.55 18685572

[ece35464-bib-0078] Steiner, F. M. , Csöcs, S. , Markó, B. , Gamisch, A. , Rinnhofer, L. , Folterbauer, C. , … Schlick‐Steiner, B. C. (2018). Molecular phylogenetics and evolution turning one into five: Integrative taxonomy uncovers complex evolution of cryptic species in the harvester ant *Messor *“*structor*”. Molecular Phylogenetics and Evolution, 127, 387–404. 10.1016/j.ympev.2018.04.005 29709692

[ece35464-bib-0079] Stork, N. E. (2018). How many species of insects and other terrestrial arthropods are there on earth? Annual Review of Ecology Evolution and Systematics, 63, 31–45. 10.1146/annurev-ento-020117-043348 28938083

[ece35464-bib-0080] Ströher, P. R. , Li, C. , & Pie, M. R. (2013). Exon‐primed intron‐crossing (EPIC) markers as a tool for ant phylogeography. Revista Brasileira de Entomologia, 57, 427–430. 10.1590/S0085-56262013005000039

[ece35464-bib-0081] Struck, T. H. , Feder, J. L. , Bendiksby, M. , Birkeland, S. , Cerca, J. , Gusarov, V. I. , … Dimitrov, D. (2018). Finding evolutionary processes hidden in cryptic species. Trends in Ecology & Evolution, 33, 153–163. 10.1016/j.tree.2017.11.007 29241941

[ece35464-bib-0082] Tajima, F. (1989). Statistical method for testing the neutral mutation hypothesis by DNA polymorphism. Genetics, 123, 585–595. 2513255 10.1093/genetics/123.3.585 PMC1203831

[ece35464-bib-0083] Tamura, K. , & Nei, M. (1993). Estimation of the number of nucleotide substitutions in the control region of mitochondrial DNA in humans and chimpanzees. Molecular Biology and Evolution, 10, 512–526. 8336541 10.1093/oxfordjournals.molbev.a040023

[ece35464-bib-0084] Thibert‐Plante, X. , & Gavrilets, S. (2013). Evolution of mate choice and the so‐called magic traits in ecological speciation. Ecology Letters, 16, 1004–1013. 10.1111/ele.12131 23782866 PMC3710524

[ece35464-bib-0085] Thomas, M. L. , & Simmons, L. W. (2008). Sexual dimorphism in cuticular hydrocarbons of the Australian field cricket *Teleogryllus oceanicus* (Orthoptera: Gryllidae). Journal of Insect Physiology, 54, 1081–1089. 10.1016/j.jinsphys.2008.04.012 18519139

[ece35464-bib-0086] Thompson, J. D. , Higgins, D. G. , & Gibson, T. J. (1994). CLUSTAL W: Improving the sensitivity of progressive multiple sequence alignment through sequence weighting, position‐specific gap penalties and weight matrix choice. Nucleic Acids Research, 22, 4673–4680. 10.1093/nar/22.22.4673 7984417 PMC308517

[ece35464-bib-0087] Thompson, J. N. , Schwind, C. , Guimarães, P. R. , & Friberg, M. (2013). Diversification through multitrait evolution in a coevolving interaction. Proceedings of the National Academy of Sciences of the United States of America, 110, 11487–11492. 10.1073/pnas.1307451110 23801764 PMC3710874

[ece35464-bib-0088] Türke, M. , Fiala, B. , Linsenmair, K. E. , & Feldhaar, H. (2010). Estimation of dispersal distances of the obligately plant‐associated ant *Crematogaster decamera* . Ecological Entomology, 35, 662–671. 10.1111/j.1365-2311.2010.01222.x

[ece35464-bib-0089] van Wilgenburg, E. , Symonds, M. R. E. , & Elgar, M. A. (2011). Evolution of cuticular hydrocarbon diversity in ants. Journal of Evolutionary Biology, 24, 1188–1198. 10.1111/j.1420-9101.2011.02248.x 21375648

[ece35464-bib-0090] van Zweden, J. S. , & d'Ettorre, P. (2010). Nestmate recognition in social insects and the role of hydrocarbons In Blomquist G. J., & Bagnères A.‐G. (Eds.), Insect hydrocarbons: Biology, biochemistry, and chemical ecology (pp. 222–243). New York, NY: Cambridge University Press.

[ece35464-bib-0091] Vantaux, A. , Dejean, A. , Dor, A. , & Orivel, J. (2007). Parasitism versus mutualism in the ant‐garden parabiosis between *Camponotus femoratus* and *Crematogaster levior* . Insectes Sociaux, 54, 95–99. 10.1007/s00040-007-0914-0

[ece35464-bib-0092] Violle, C. , Nemergut, D. R. , Pu, Z. , & Jiang, L. (2011). Phylogenetic limiting similarity and competitive exclusion. Ecology Letters, 14, 782–787. 10.1111/j.1461-0248.2011.01644.x 21672121

[ece35464-bib-0093] Vodă, R. , Dapporto, L. , Dincă, V. , & Vila, R. (2015). Why do cryptic species tend not to co‐occur? A case study on two cryptic pairs of butterflies. PLoS ONE, 10, e0117802 10.1371/journal.pone.0117802 25692577 PMC4334660

[ece35464-bib-0094] Wickham, H. (2016). ggplot2: Elegant graphics for data analysis, 2nd ed. New York, NY: Springer‐Verlag.

[ece35464-bib-0095] Wolak, M. E. , Fairbairn, D. J. , & Paulsen, Y. R. (2012). Guidelines for estimating repeatability. Methods in Ecology and Evolution, 3, 129–137. 10.1111/j.2041-210X.2011.00125.x

