# Supplementary material for: Cuticular hydrocarbons as potential mediators of cryptic species divergence in a mutualistic ant association
Source: Ecol Evol. 2019 Jul 21;9(16):9160–76. doi: 10.1002/ece3.5464 (PMC6706187; doi:10.1002/ece3.5464)
Supplement: Supplementary file 1 [file ECE3-9-9160-s001.pdf]

Supporting figures

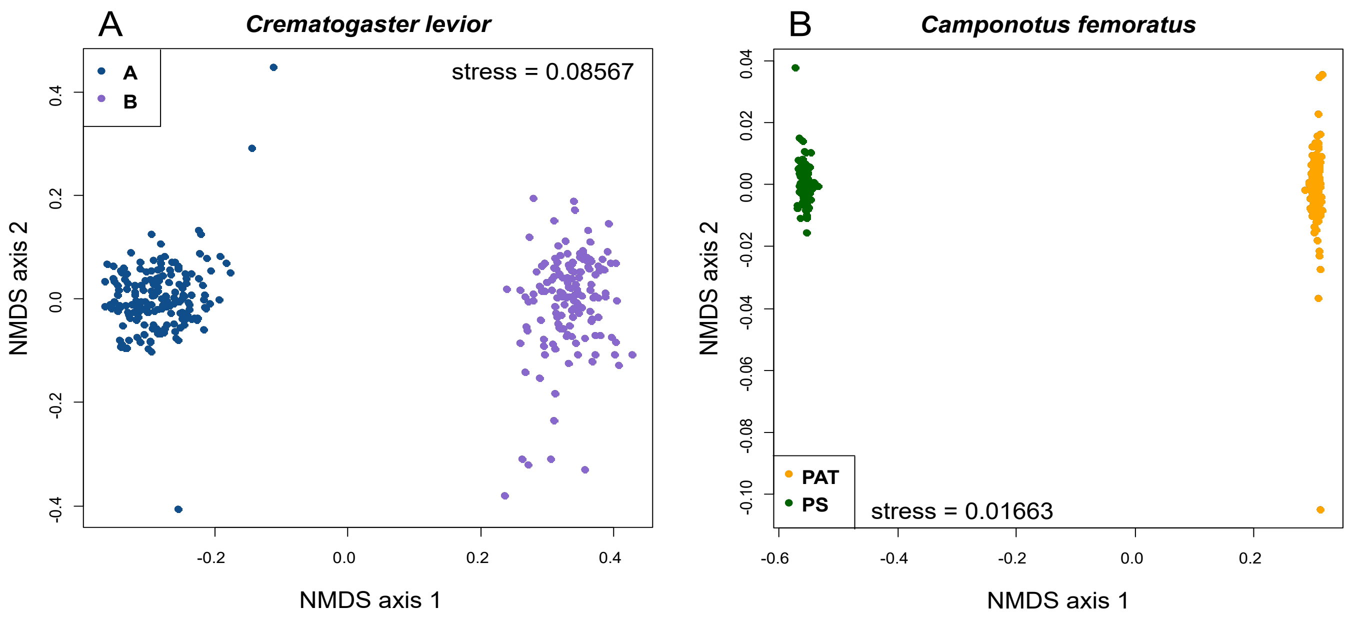

Figure S1: Non-metric multidimensional scaling (NMDS) ordinations of the CHC profiles of the cryptic *Cr. levior* and *Ca. femoratus* species. (A) Shows an NMDS ordination of *Cr. levior* CHC profiles (*Cr. levior* A: blue; *Cr. levior* B: purple). Each dot represents one colony. (B) Shows a similar ordination for *Ca. femoratus* CHCs (*Ca. femoratus* PAT: yellow; *Ca. femoratus* PS: green).

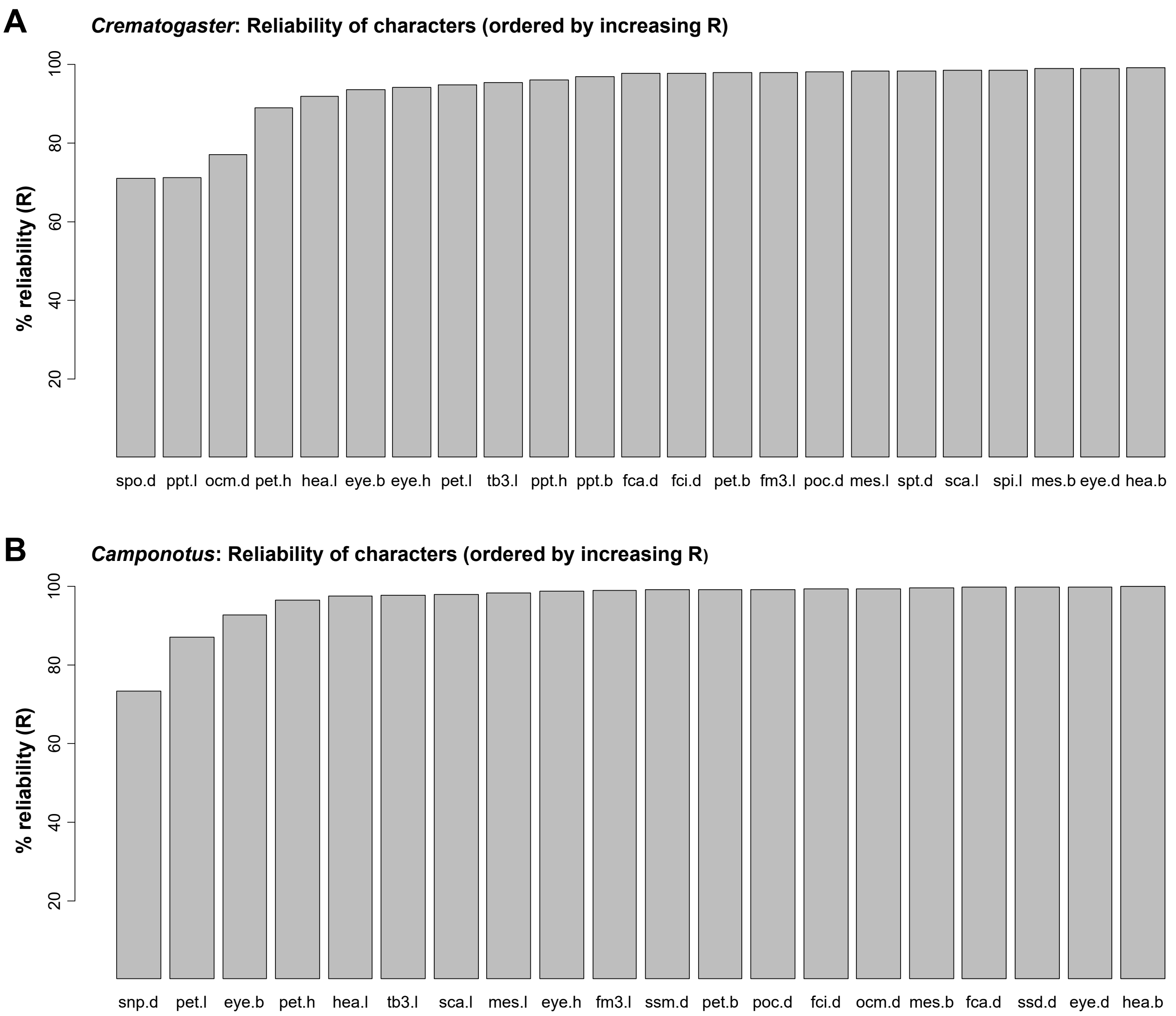

Figure S2: Reliability of morphological characters. The barplots show the reliability (R) of characters measured in *Crematogaster* (A) and *Camponotus* (B). Characters with a reliability < 85% were not included into the multivariate ratio analysis.

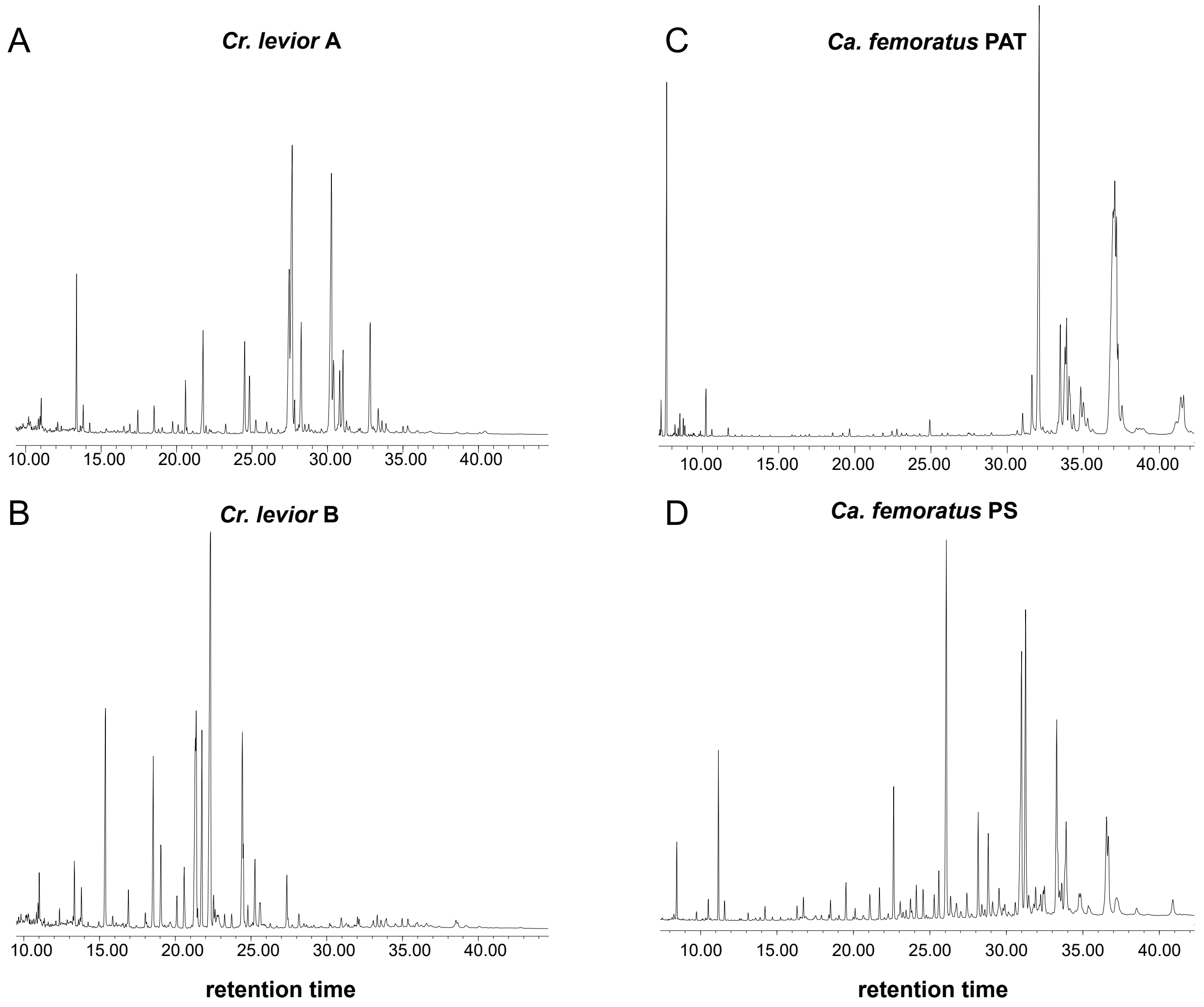

Figure S3: Representative chromatograms of the cuticular hydrocarbon profiles of the cryptic species. (A+B) Show chromatograms of the CHC profiles of *Cr. levior* A and B. (C+D) Show chromatograms of the CHC profiles of *Ca. femoratus* PAT and PS.

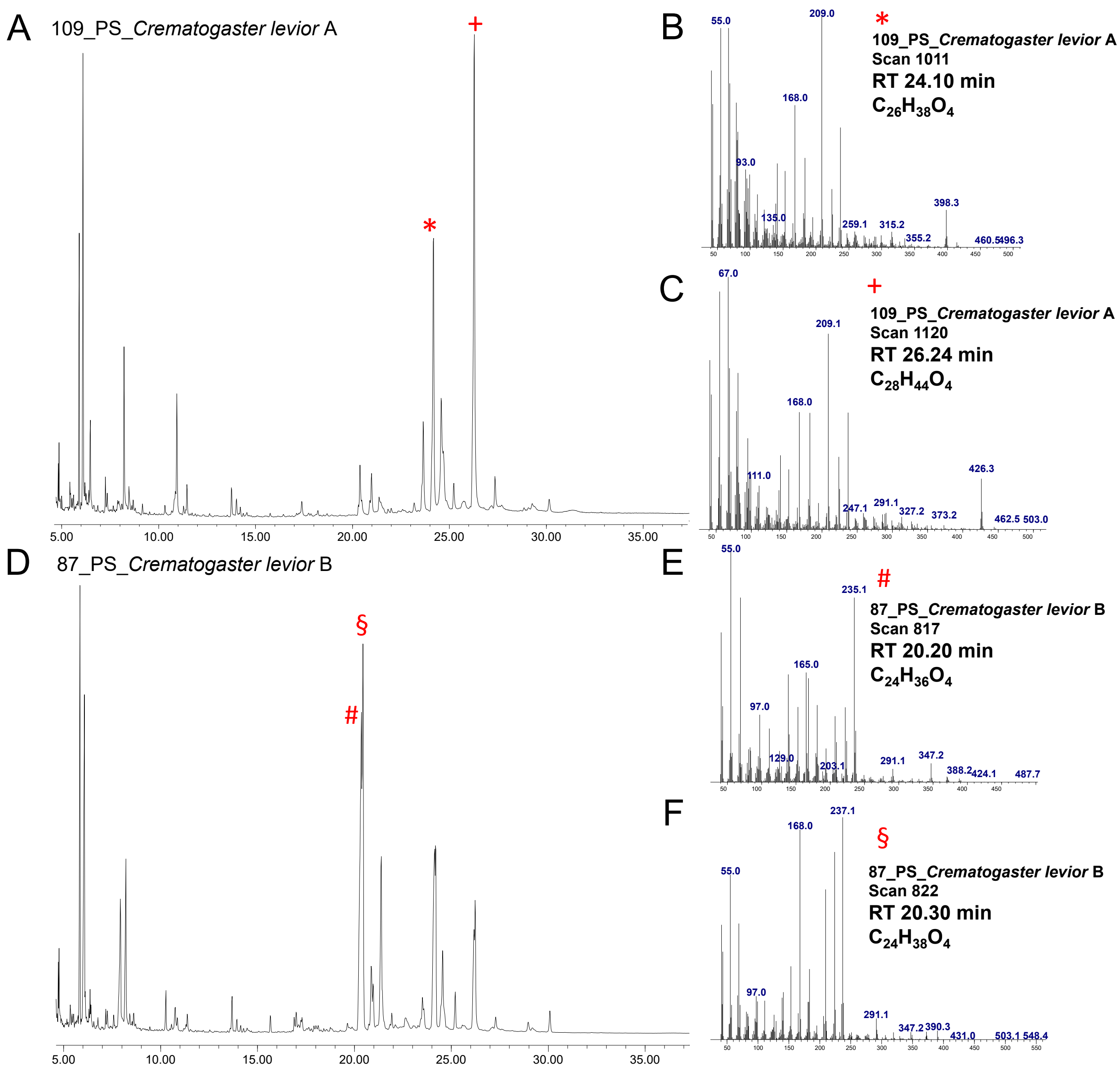

Figure S4: Representative chromatograms of the polar substances of *Cr. levior* A and B and mass spectra of the most abundant substances. (A-C) Show a representative chromatogram of the polar substances of *Cr. levior* A (A), and the mass spectra of the two most common substances in this species (B+C). (D-F) Show a representative chromatogram of the polar substances of *Cr. levior* B (D), and the mass spectra of the two most common substances in this species (E+F).

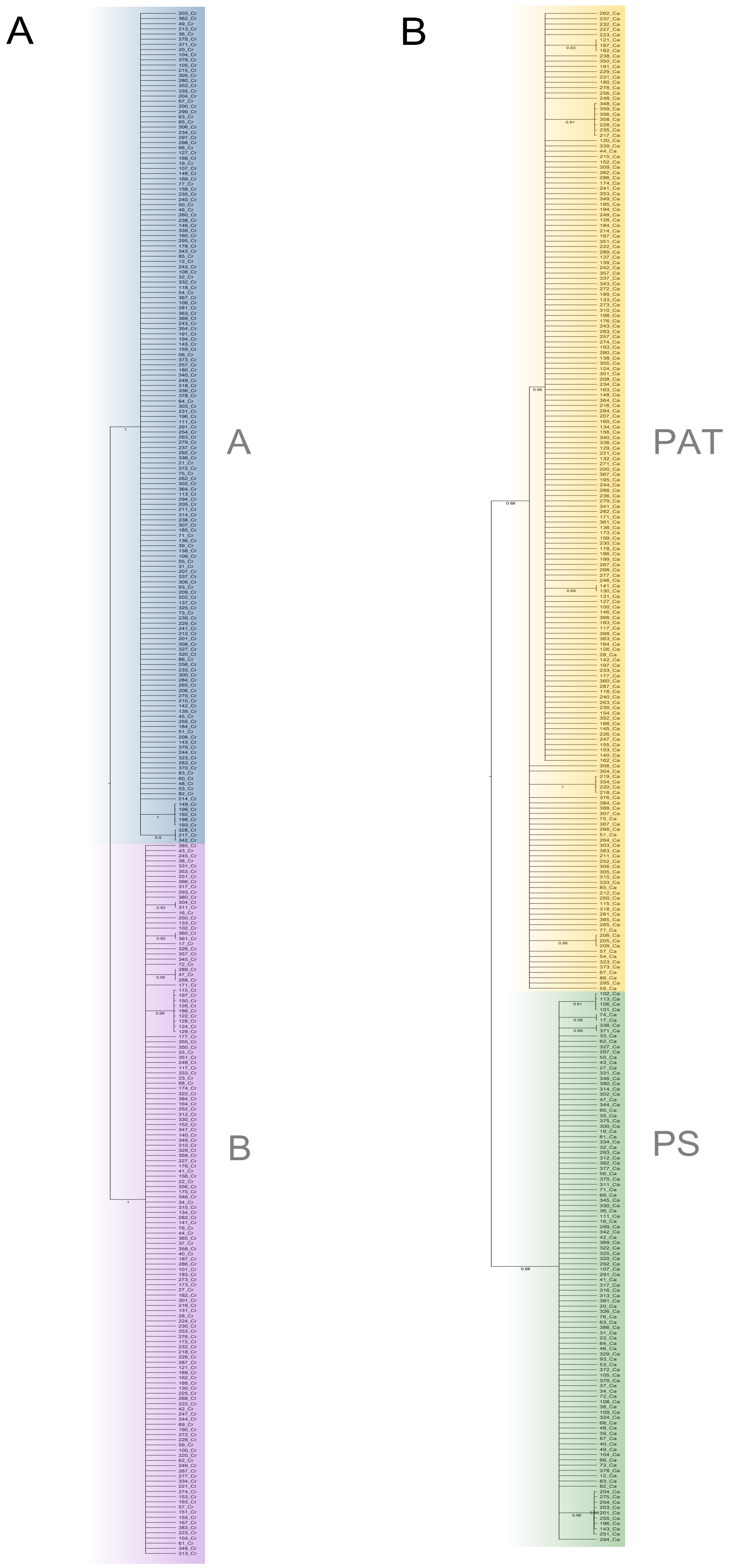

Figure S5: MrBayes Phylogeny of mitochondrial COI of (A) *Crematogaster levior s*pecies and (B) *Camponotus femoratus* species.

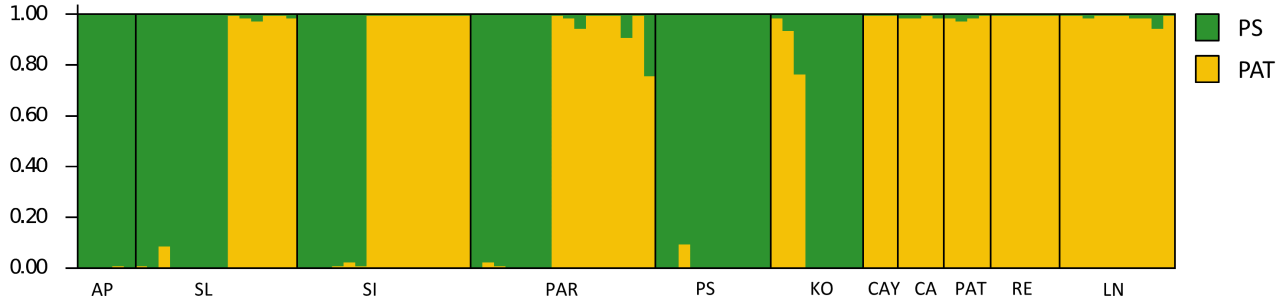

Figure S6: STRUCTURE analysis of 93 individuals of *Camponotus femoratus*, for which all four nuclear loci were sequenced successfully. For abbreviations of population names refer to Table 1.
