# Supplementary material for: Cuticular hydrocarbons as potential mediators of cryptic species divergence in a mutualistic ant association
Source: Ecol Evol. 2019 Jul 21;9(16):9160–76. doi: 10.1002/ece3.5464 (PMC6706187; doi:10.1002/ece3.5464)
Supplement: Supplementary file 2 [file ECE3-9-9160-s002.docx]

**Supporting Tables**

**Table S1: Reliability of body measures.** The table shows the variable codes and names, the reliability R as well as the lower and upper 95 % confidence intervals (LCI and UCI). For definitions of measures see our Dryad data (doi:10.5061/dryad.m7ks1g8).

| **variable code** | **variable name** | **R** | **LCI** | **UCI** |
| --- | --- | --- | --- | --- |
| ***Crematogaster*** | | | | |
| spo.d | Min. spine distance | 70.99 | 48.40 | 82.94 |
| ppt.l | Postpetiole length | 71.30 | 48.91 | 83.13 |
| ocm.d | Ocular-malar-distance | 77.16 | 58.77 | 86.67 |
| pet.h | Petiole height | 89.07 | 79.68 | 93.71 |
| hea.l | Head length | 91.81 | 84.68 | 95.31 |
| eye.b | Eye breadth | 93.64 | 88.05 | 96.36 |
| eye.h | Eye length | 94.19 | 89.07 | 96.68 |
| pet.l | Petiole length | 94.91 | 90.40 | 97.09 |
| tb3.l | Tibia length | 95.47 | 91.44 | 97.41 |
| ppt.h | Postpetiole height | 96.04 | 92.52 | 97.74 |
| ppt.b | Postpetiole breadth | 96.97 | 94.25 | 98.27 |
| fca.d | Max. distance of frontal lobes | 97.77 | 95.77 | 98.73 |
| fci.d | Min. distance of frontal lobes | 97.81 | 95.85 | 98.76 |
| pet.b | Petiole breadth | 97.91 | 96.04 | 98.81 |
| fm3.l | Femur length | 98.01 | 96.23 | 98.87 |
| poc.d | Postocular-distance | 98.11 | 96.41 | 98.93 |
| mes.l | Mesosoma length | 98.29 | 96.75 | 99.03 |
| spt.d | Max. spine distance | 98.33 | 96.83 | 99.05 |
| sca.l | Scape length of the antennae | 98.56 | 97.26 | 99.18 |
| spi.l | Spine length | 98.57 | 97.29 | 99.19 |
| mes.b | Mesosoma breadth | 98.97 | 98.03 | 99.41 |
| eye.d | Min. head breadth | 99.00 | 98.09 | 99.43 |
| hea.b | Max. head breadth | 99.16 | 98.40 | 99.52 |
| ***Camponotus*** | | | | |
| snp.d | Stigma-spine of petiole distance | 73.32 | 52.22 | 84.55 |
| pet.l | Petiole length | 87.15 | 76.12 | 92.70 |
| eye.b | Eye breadth | 92.73 | 86.27 | 95.90 |
| pet.h | Petiole height | 96.45 | 93.23 | 98.01 |
| hea.l | Head length | 97.52 | 95.25 | 98.61 |
| tb3.l | Tibia length | 97.68 | 95.57 | 98.70 |
| sca.l | Scape length of antennae | 97.91 | 96.00 | 98.83 |
| mes.l | Mesosoma length | 98.36 | 96.86 | 99.08 |
| eye.h | Eye length | 98.69 | 97.48 | 99.27 |
| fm3.l | Femur length | 98.92 | 97.92 | 99.39 |
| ssm.d | Stigma distance | 99.23 | 98.52 | 99.57 |
| pet.b | Petiole breadth | 99.28 | 98.61 | 99.60 |
| poc.d | Postocular-distance | 99.28 | 98.62 | 99.60 |
| fci.d | Min. distance frontal lobes | 99.41 | 98.86 | 99.67 |
| ocm.d | Ocular-malar-distance | 99.45 | 98.94 | 99.69 |
| mes.b | Mesosoma breadth | 99.73 | 99.49 | 99.85 |
| fca.d | Max. distance frontal lobes | 99.74 | 99.50 | 99.85 |
| ssd.d | Mesosoma stigma distance | 99.76 | 99.54 | 99.87 |
| eye.d | Min. head breadth | 99.89 | 99.79 | 99.94 |
| hea.b | Max. head breadth | 99.96 | 99.92 | 99.98 |

**Table S2: PCR mastermix and sequencing reaction.**

| **PCR mastermix** | **Volume** | **Sequencing reaction** | **Volume** |
| --- | --- | --- | --- |
| 10x Buffer B (molegene) | 1.0 µl | Big Dye Terminator Mix | 0.16 µl |
| MgCL_2_ 25mM (molegene) | 1.0 µl | 5x Buffer B (molegene) | 1.84 µl |
| dNTP-Mix 2mM each (molegene) | 0.1 µl | ddH_2_O | 6.50 µl |
| Primer 10 pmol/µl) | 0.2 µl of each primer | Primer (10 pmol/µl) | 0.5 µl of each primer |
| Taq-Polymerase (molegene) | 0.1 µl | PCR product | 1.0µl |
| ddH_2_O | 6.5 µl |  |  |

**Table S3: Overview of all test primer pairs.** *(Ströher et al. 2013)*

| **Primer** | **Primer length** | **Primer sequence** |  |
| --- | --- | --- | --- |
| ant. 1F | 27 | CCTTCGTGCCTAYGAGAATAGYGTTAC | Resulting sequences were variable enough for further analyses |
| ant. 1R | 21 | AACGACGTCSGACGGTTCCAT |  |
| ant. 389F | 21 | ACGGACCCCACATTGAGAAGAAC |  |
| ant. 389R | 21 | CYTTACCCACCTCCTCCACCA |  |
| ant. 1087 F | 21 | ACCAGCAGAGGCTGGACGTGA |  |
| ant. 1087 R | 27 | GCCAAGTTGATTGTGTACGAACTTTCT |  |
| ant. 1401F | 22 | GYAGGAAGGACGCTCTTAATCT |  |
| ant. 1401R | 26 | AAGCTTATCTCTAGGAAACTCCCATC |  |
| ant. 1225 F | 26 | TAATACRACTGAAGAGAGACCAGGAG | Not used for further analyses as resulting sequences were either not variable or did not amplify in PCR |
| ant. 1225 R | 27 | GACTAGATCCTAAGCTAGAGAGRCTGG |  |
| ant. 1281 F | 23 | GACGCAGGTTGYAACGAAATCAC |  |
| ant. 1281 R | 24 | GCCRCTAATATCCAGCTTCACGAG |  |
| ant. 384 F | 27 | TAGTAGTCGAAGGAGTCATACCAAAGG |  |
| ant. 384 R | 20 | TGYGTGTTCGATGCCGTTGA |  |
| ant. 965 F | 24 | AGTTCAAGGTTCACCGGTGCCTAA |  |
| ant. 965 R | 25 | GAGAAGGYGAAYTTAAAGACTGATG |  |
| ant. 1503F | 21 | GRTTYGCCTTCCAGGAGATCA |  |
| ant. 1503R | 23 | AAGTAGTCCAGGCAGAACCACAC |  |
| ant. 202F | 26 | CCYATCAACTCTGTTAATATCGAACG |  |
| ant. 202R | 22 | GACACAATGTTGGAAGCCCTTG |  |
| ant. 263F | 27 | GACTAGCTCAGAATCACACTCTTCCAC |  |
| ant. 263R | 24 | GTTGTTTTGGWGGCAATATTGGAG |  |
| ant. 346F | 23 | GTGGTCCACCATCCGTKGGATCT |  |
| ant. 346R | 26 | GGATTGTTTTGTGTAATCTGCGTTCG |  |
| ant. 505F | 24 | CCTCAGATGAAGTTYCGAGTTCC |  |
| ant. 505R | 26 | TAAYCCGRACACCCTCACTTTATACG |  |
| ant. 839F | 25 | CAATGGCGATTTACAACGAATTTCT |  |
| ant. 839R | 22 | CAGGCANAGCAGCAATGTGACG |  |

**Table S4: Tajima’s D statistics.** Given are values within all sampled populations of *Crematogaster levior* B and *Camponotus femoratus* PS and PAT. Bold characters indicate statistical significance (p < 0.05) based on a permutation test. *Crematogaster levior* A is not shown as there was only a single haplotype found at all locations.

|  | ***Cr. levior* B** | | ***Ca. femoratus* PS** | | ***Ca. femoratus* PAT** | |
| --- | --- | --- | --- | --- | --- | --- |
|  | Tajima's D | p | Tajima's D | p | Tajima's D | p |
| *AP* | 0 | 1 | 0 | 1 | 0 | 1 |
| *SL* | 0 | 1 | **-1.513** | **0.033** | **-1.486** | **0.04** |
| *SI* | -1.088 | 0.189 | 0 | 1 | 1.284 | 0.888 |
| *PS* | 0 | 1 | 0.545 | 0.764 | 0 | 1 |
| *PAR* | -1.149 | 0.161 | -1.142 | 0.145 | **-2.072** | **0.003** |
| *LN* | 0.713 | 0.869 | 0 | 1 | **-2.107** | **0.002** |
| *KO* | 1.225 | 0.943 | -0.817 | 0.321 | 0 | 1 |
| *MT* | - | - | 0 | 1 | 0 | 1 |
| *CA* | 0 | 1 | - | - | 0.944 | 0.843 |
| *CAY* | 0 | 1 | - | - | 2.125 | 0.986 |
| *RE* | - | - | 0 | 1 | 2.192 | 0.993 |
| *PAT* | -0.774 | 0.218 | - | - | -0.657 | 0.297 |
